# CE-ICP-MS Investigation of the Carbonate Complexation of Am(III), Th(IV), Np(V), and U(VI) and the Influence of Alkali Cations

**DOI:** 10.1021/acs.inorgchem.5c02509

**Published:** 2025-10-16

**Authors:** Janik Lohmann, Stefanie Isabella Demel, Justus Carl Sander, Tobias Reich

**Affiliations:** 9182 Johannes Gutenberg-Universität Mainz, Department of Chemistry-Nuclear Chemistry, 55099 Mainz, Germany

## Abstract

Capillary electrophoresis inductively coupled plasma mass spectrometry (CE-ICP-MS) was used to investigate the carbonate complexation of Am­(III), Th­(IV), Np­(V), and U­(VI) at environmentally relevant concentrations. Experiments were conducted at an ionic strength of 0.33 M in different alkali chloride solutions (Li^+^, Na^+^, and K^+^). Formation constants for the three successive actinide carbonate complexes were determined for Am­(III), Np­(V), and U­(VI). For Th­(IV), the CE-ICP-MS measurements posed some challenges. The complexation of U­(VI) with carbonate was determined to be stronger than previously assumed. A strong influence of alkali cations on the electrophoretic mobility μ of highly negatively charged actinide carbonate complexes was observed and attributed to the association of cations to those complexes. The electrophoretic mobility of negatively charged An-CO_3_ complexes was found to follow the trend |μ_Li_| ≤ |μ_Na_| < |μ_K_|. Furthermore, the reaction of UO_2_(CO_3_)_3_^4–^ with different alkali cations (Me^+^ = Li^+^–Cs^+^) was studied. Complex formation constants for MeUO_2_(CO_3_)_3_^3–^ and Me_2_UO_2_(CO_3_)_3_^2–^ for all investigated cations were determined.

## Graphical abstract

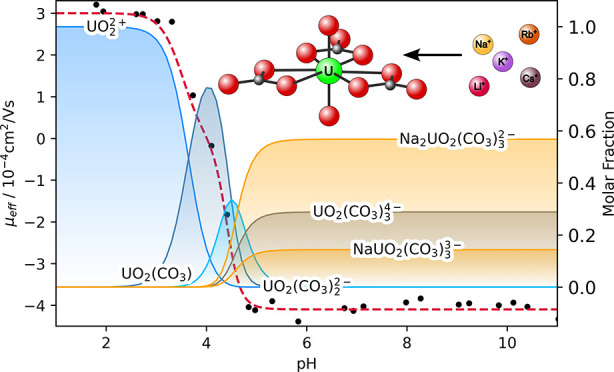

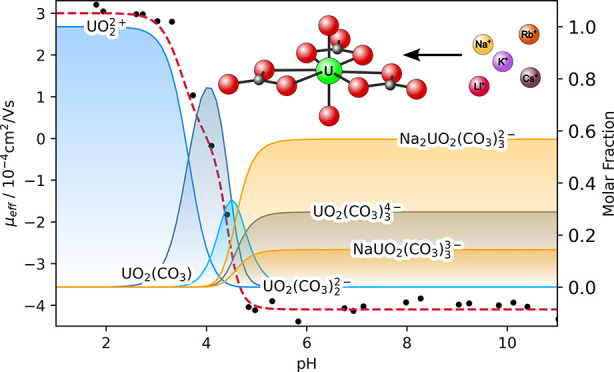

## 1 Introduction

For the safety analysis of a high-level nuclear waste repository, it is necessary to investigate the behavior of the long-lived actinides under environmentally relevant conditions. Processes like actinide diffusion and sorption are strongly dependent on the actinide species present.^1^ Therefore, it is important to have a thorough understanding of the complexation of actinides, including thermodynamic constants and molecular structures. The Nuclear Energy Agency (NEA) has extensively compiled and reviewed thermodynamic data with their Thermochemical Database (TDB) Project.^2–5^ A wide range of experimental methods are needed to produce satisfying thermodynamic data. Experiments at trace-level concentrations, which better reflect the environmentally relevant conditions, are of particular interest.

Due to its ubiquitous nature, carbonate occurs in all surface waters. But also in pore waters underground, significant carbonate concentrations can be present. Opalinus clay pore water for example contains 5 × 10^–4^ M carbonate.^6^ Carbonate is, together with hydroxide, one of the most environmentally relevant ligands regarding actinides and plays a significant role in their mobilization.

Many of the corresponding complex formation constants for the actinides up to Cm are described in literature. The complexation of Am­(III) with carbonate was previously studied mainly by solubility experiments^7^ or optical methods.^8^ In some TLRFS experiments,^9,10^ Cm­(III) was used as an analog for Am­(III). Most studies agree on the formation of three successive An­(III) carbonate complexes. For Cm­(III), a fourth complex Cm­(CO_3_)_4_^5–^ has been discussed.^10^ This complex has not been observed for Am­(III) yet.^5^ Under a high CO_2_ partial pressure >1 bar, the formation of a Cm­(III) bicarbonate complex CmHCO_3_^2+^ has been observed by Fanghänel et al.^9^ While the use of Cm­(III) enables experiments at trace concentrations, experiments using Am(III) were done at the solubility limit of Am­(III).^5^

Studies investigating the complexation of Th­(IV) with carbonates were summarized in the NEA review by Rand et al.^4^ Th­(IV) has a strong affinity for carbonate as well as hydroxide anions. With its high coordination number, about 20 different binary and ternary complexes are possible.^11^ Due to the complexity of the system, there are some inconsistencies in literature concerning the dominating complexes. Altmaier et al.^12^ describe the ternary Th­(OH)­(CO_3_)_4_^5–^ and Th(OH)_2_(CO_3_)_2_^2–^ complexes as the most important ones, while Östhols et al.^13^ describe the Th­(CO_3_)_5_^6–^ complex as dominating at higher pH values. In both studies, the solubility of Th­(IV) hydrous oxide was investigated in carbonate solution resulting in Th­(IV) concentrations up to 1 × 10^–3^ M.

The Np­(V) carbonate system has been reported extensively in literature.^2^ Topin et al.^14^ and Aupiais et al.^15^ successfully studied the system using a coupling between capillary electrophoresis and inductively coupled plasma mass spectrometry (CE-ICP-MS) at environmentally relevant concentrations (1 × 10^–7^ M). For Np­(V) three successive carbonate complexes are known with NpO_2_(CO_3_)_3_^5–^ being the limiting complex. The formation of ternary Np­(V)–CO_3_–OH complexes has been discussed in the review done by Lemire et al.,^2^ and a value for the formation of the NpO_2_(CO_3_)_2_OH^4–^ complex was selected.

The complexation of U­(VI) with carbonate was studied mostly in solubility experiments^3,16–18^ with concentrations above 1 × 10^–5^ M. Three successive U­(VI) carbonate complexes were identified along with the polynuclear complex (UO_2_)_3_(CO_3_)_6_^6–^. The polynuclear complex becomes irrelevant at lower U­(VI) concentrations.

Except for Cm­(III) and Np­(V) most complexation studies were carried out at significantly higher actinide concentrations than expected in the case of a release of actinides from a waste repository into the environment. CE-ICP-MS is an invaluable tool to investigate the complexation behavior of actinides at trace concentrations.^14,15,19,20^ In this work, the complexation behavior of ^241^Am­(III), ^232^Th­(IV), ^237^Np­(V), and ^238^U­(VI) at trace-level with carbonate was investigated by CE-ICP-MS with the aim of determining complex formation constants. The separation capability of the CE, based on charge-to-radius ratio, combined with the mass selectivity of the ICP-MS enabled simultaneous investigations of all four actinides.

In previous CE-ICP-MS studies,^14,15,20^ an influence of alkali cation on the electrophoretic mobility of negatively charged carbonate complexes was observed. To further study this effect, in this work the influence of alkali cations Li^+^, Na^+^, K^+^, Rb^+^, and Cs^+^ on the carbonate complexation was investigated. The charge sensitivity of CE-ICP-MS is ideal for the investigation of potential association reactions.

## 2 Experimental Section

### 2.1 Determination of Complexation Constants by Capillary Electrophoresis

The determination of complex formation constants by CE is described in detail by Willberger et al.^19^ From the CE measurements, migration times of the actinides and the neutral marker for the electroosmotic flow (EOF) were determined. The effective electrophoretic mobility μ_eff_ can be calculated by eq 1 with the migration time of the actinide *t*_An_, the migration time of a neutral marker *t*_EOF_, indicating the EOF, the effective length *l* of the capillary and the applied voltage *U*.

1 $$\mu_{\mathrm{eff}}=\frac{l^{2}}{U}\left(\frac{1}{t_{\mathrm{An}}}-\frac{1}{t_{\mathrm{EOF}}}\right)$$

For the carbonate complexation, the exchange between the species present in solution occurs fast in the time frame of the electrophoretic separation, so only one peak with the average mobility of all species present is measured. The measured effective mobility μ_eff_ is therefore made up of the proportions α_*i*_ of these species and their corresponding mobilities μ_*i*_ (eq 2)­

2 $$\mu_{\mathrm{eff}}=\sum_{i=0}^{N}\alpha_{i}\mu_{i}$$

The equilibria of actinides Am^3+^ and AnO_2_^z^ (NpO_2_^+^ and UO_2_^2+^) with carbonate shown in eqs 3 through 6 are assumed. For Am­(III), Np­(V), and U­(VI), the limiting binary carbonate complex is assumed to be the 1:3 complex.

3 $${\mathrm{Am}}^{3+}+iCO_{3}^{2-}⇌\mathrm{Am}(CO_{3}{)}_{i}^{3-2i}$$

4 $$\mathrm{An}O_{2}^{z}+iCO_{3}^{2-}⇌\mathrm{An}O_{2}(CO_{3}{)}_{i}^{z-2i}$$

In addition, the AmHCO_3_^2+^ species, based on the analogy with CmHCO_3_^2+^ observed by Fanghänel et al.,^9^ (eq 5) and the NpO_2_(CO_3_)_2_OH^4–^ complex (eq 6) are expected to form under the experimental conditions.

5 $${\mathrm{Am}}^{3+}+CO_{3}^{2-}+H^{+}⇌\mathrm{AmHC}O_{3}^{2+}$$

6 $$\mathrm{Np}O_{2}^{+}+2CO_{3}^{2-}+H_{2}O⇌\mathrm{Np}O_{2}(CO_{3}{)}_{2}{\mathrm{OH}}^{4-}+H^{+}$$

The cumulative complex formation constants β corresponding to eqs 3 through 6 are defined in eqs 7 through 10. The square brackets indicate molar concentration of the reactants.

7 $$\beta_{i}=\frac{\left[{\mathrm{Am}{\left(CO_{3}\right)}_{i}}^{3-2i}\right]}{[{\mathrm{Am}}^{3+}]{\left[CO_{3}^{2-}\right]}^{i}}$$

8 $$\beta_{1,H}=\frac{\left[{\mathrm{Am}\left(\mathrm{HC}O_{3}\right)}^{2+}\right]}{[{\mathrm{Am}}^{3+}]\left[CO_{3}^{2-}\right]\left[H^{+}\right]}$$

9 $$\beta_{i}=\frac{\left[{\mathrm{An}{O_{2}\left(CO_{3}\right)}_{i}}^{z-2i}\right]}{[{\mathrm{An}O_{2}}^{z}]{\left[CO_{3}^{2-}\right]}^{i}}$$

10 $$\beta_{2,\mathrm{OH}}=\frac{[{\mathrm{Np}{O_{2}\left(CO_{3}\right)}_{2}\mathrm{OH}}^{4-}]\left[H^{+}\right]}{[{\mathrm{Np}O_{2}}^{+}]{\left[CO_{3}^{2-}\right]}^{2}}$$

Using eq 9 for example, the proportions α_*i*_ with *i* = 1, 2, 3 of the carbonate species present in solution for U­(VI) can be expressed by eq 11.

11 $$\alpha_{i}=\frac{\beta_{i}{\left[CO_{3}^{2-}\right]}^{i}}{1+\sum_{x=1}^{3}\beta_{x}{\left[CO_{3}^{2-}\right]}^{x}}$$

Combining eqs 2 and 11, eq 12 is obtained, with which the complex formation constants can be determined from the measured electrophoretic mobility μ_eff_ in relation to the free carbonate concentration [CO_3_^2–^]. For Am­(III) and Np­(V) the corresponding equations are shown in the Supporting Information (eqs S1 and S2, Supporting Information)

12 $$\mu_{\mathrm{eff}}=\frac{\mu_{0}+\sum_{i=1}^{3}{\mu_{i}\beta }_{i}{\left[{\mathrm{CO}}_{3}^{2-}\right]}^{i}}{1+\sum_{i=1}^{3}\beta_{i}{\left[{\mathrm{CO}}_{3}^{2-}\right]}^{i}}$$

The electrophoretic mobility of the actinides in absence of carbonate μ_0_ is made up of the mobilities of the free actinide as well as the corresponding chloro-complexes. Because chloride concentrations did not change throughout the experiments, the ratio between the free actinide and the actinide chloro complex stays the same and μ_0_ can substitute the mobility of the free actinide. Therefore, chloro-complexes are not considered further.

The concentration of free carbonate [CO_3_^2–^] as a function of its initial concentration *c*_0_ and pH can be calculated using eq 13.

13 $$\left[CO_{3}^{2-}\right]=\frac{c_{0}}{{1+10}^{\left(pK_{a2}-\mathrm{pH}\right)}+10^{\left(pK_{a1}+pK_{a2}-2\mathrm{pH}\right)}}$$

The p*K*_a_ ^5^ values of H_2_CO_3_ were adjusted to an ionic strength of 0.33 M (converted to molality) using the specific ion interaction theory (SIT) and the ion interaction coefficients of the corresponding alkali cation^21^ (summarized in SI Table S6). The p*K*_a_ values are shown in Table 1. For Li^+^ no ion interaction coefficients were found, so the p*K*_a_ values were adjusted using data for Na^+^.

**Table 1:** p*K*_a_ Values of Carbonic Acid Adjusted to *I* = 0.335 m for Li^+^, *I* = 0.333 m for Na^+^, and *I* = 0.335 m for K^+^

cation	p*K*_a1_^*I*^	p*K*_a2_^*I*^
Li^+^, Na^+^	6.08	9.71
K^+^	6.06	9.77

### 2.2 CE-ICP-MS

All CE measurements were performed using an Agilent 7100 CE system (Agilent Technologies, Waldbronn, Germany) hyphenated to an Agilent 7900 ICP-MS (Agilent Technologies, Wiesental, Germany). This coupling was realized via a MiraMist CE nebulizer (Burgener Research, Mississauga, Canada) and a Scott-type spray chamber (AHS Analysentechnik, Tübingen, Germany). To aid the aerosol formation, a makeup electrolyte containing 1.25% HNO_3_, 10% ethanol and 5 ppb ^7^Li, ^24^ Mg, ^59^Co, ^89^Y, ^140^Ce, and ^205^Tl acting as internal standards was added via the peristaltic pump of the ICP-MS with a flow rate of 15 μL/min. 2‑Bromopropane was added to the samples to mark the electroosmotic flow by detecting ^79^Br by ICP-MS.

For the CE experiments, a fused silica capillary with 50 μm inner diameter and 50 cm length was used (Polymicro Technologies, Phoenix, Arizona, USA). At the start of each experimental series, the capillary was preconditioned using Milli-Q water, 0.1 M HCl, and 0.1 M NaOH. Before each measurement, the capillary was flushed with a background electrolyte (BGE) with the same composition as the sample, but without actinides. Then, 15 nL of the sample were injected hydrodynamically at 100 mbar for 5 s. The capillary was placed back into the BGE and a voltage of 10 kV and a pressure of 60 mbar were applied. The temperature was kept at 25.0 ± 0.1 °C using the internal air cooling of the CE device as well as a custom build enclosure for the hyphenation.

The ICP-MS was used in time-resolved analysis mode for detection with a dwell time of 100 ms and a plasma at 1550 W. The carrier gas flow rate was set to 1 L/min and no makeup gas was used. The detected masses were ^79^Br, ^89^Y, ^127^I, ^232^Th, ^237^Np, ^238^U, and ^241^Am. In most cases, the peak search of the MassHunter 5.1 software (Agilent Technologies, Santa Clara, California, USA) was used to extract the migration times. For ambiguous peaks, the migration time was selected manually.

### 2.3 Reagents

**Caution!** ^232^Th, ^237^Np, ^238^U, and ^241^Am are radioactive elements and require special precautions as well as radiation protection.

All chemicals used were of analytical grade or better. Milli-Q water was used throughout all experiments (18.2 MΩcm, Synergy Millipore water system, Millipore GmbH, Schwalbach, Germany). To prevent clogging, all solutions used were filtered through syringe filters (0.2 μm, Nalgene, Rochester, New York, USA). A list of all suppliers for the chemicals used can be found in SI (Table S5).

An ^241^Am­(III) stock solution was prepared by evaporating an in-house ^241^Am solution to dryness and redissolving in 0.1 M HClO_4_. For the ^237^Np­(V) stock solution, an in-house ^237^Np solution was evaporated until near dryness and redissolved in 1 M HClO_4_. This process was repeated three times to yield a Np­(VI) solution. In the last step, the ^237^Np was dissolved in 0.1 M HClO_4_ and NaNO_2_ was added to reduce the Np­(VI) to Np­(V). The oxidation state was confirmed by UV–vis spectroscopy (Tidas 100, J&M Analytik AG, Essingen, Germany). The concentrations of the ^241^Am and ^237^Np solutions were determined by γ-ray spectroscopy (^241^Am at 59.5 keV, ^237^Np at 86.5 keV) using a high-purity germanium detector (GMX-13280-S, ORTEC, Oak Ridge, Tennessee, USA) and the Canberra InSpector 2000DSP Portable Workstation (Model IN2K, Canberra Industries Inc., Meriden, Connecticut, USA). For the ^232^Th­(IV) and ^238^U­(VI) stock solutions, ICP-MS standards (^232^Th: Accu Trace, Accu Standard, New Haven, Connecticut, USA, ^238^U: SPEX Certiprep, Metuchen, Massachusetts, USA) of known concentrations were evaporated and redissolved in 0.1 M HClO_4_. All stock solutions were combined to produce an actinide cocktail with [^241^Am] = 3 × 10^–5^ M, [^232^Th] = 1 × 10^–3^ M, [^237^Np] = 2 × 10^–4^ M, and [^238^ *U*] = 2 × 10^–4^ M in 0.1 M HClO_4_. If not stated otherwise, this actinide cocktail was used throughout all experiments.

### 2.4 Sample Preparations

For each BGE, the pH value of a 0.1 M Me_2_CO_3_ solution (Me = Li, Na, K) was varied between pH 2 and pH 11 using HCl. The ionic strength was raised to *I* = 0.33 M using the corresponding alkali chloride salt. As buffers, 0.05 M MES (2-(*N*-morpholino)­ethanesulfonic acid) was added between pH 4 and pH 7, 0.05 M HEPES (4-(2-hydroxyethyl)-1-piperazineethanesulfonic acid) between pH 7 and pH 8, and 0.05 M CHES (*N*-cyclohexyl-2-aminoethanesulfonic acid) between pH 8 and pH 10. No buffer was added below pH 4 and above pH 10. The pH values were measured with an inoLab pH 720 meter (WTW a Xylem brand, Weilheim, Germany) and a BlueLine 16 pH microelectrode (SI Analytics a Xylem brand, Mainz, Germany) filled with 3 M NaCl solution. The device was calibrated with reference buffer solutions at pH 4.01, pH 6.87, and pH 9.18. The detailed composition of each sample can be found in SI (Tables S7, S9, S11, S13). To flush the capillary prior to the CE measurements, 200 μL of each BGE were added to glass vials and capped off using polyethylene snap caps.

The actinide cocktail was diluted using an aliquot of the BGEs to produce the following concentrations: [^241^Am] = 3 × 10^–8^ M, [^232^Th] = 1 × 10^–6^ M, [^237^Np] = 2 × 10^–7^ M, and [^238^ *U*] = 2 × 10^–7^ M.

The pH values were remeasured upon actinide addition and readjusted, if necessary. In most samples, no significant change in pH was observed. Directly before each CE-measurement, the pH value was checked again. A portion of 200 μL of the actinide containing sample was transferred to glass vials along with 1 μL of 2-bromopropane as a marker for the EOF.

For the determination of the complex formation constants for the ternary alkali-actinide-carbonate complexes, only U­(VI) was chosen for its wide range in pH and carbonate concentration where the UO_2_(CO_3_)_3_^4–^ complex predominates. For this measurement series, BGEs with ionic strengths between *I* = 0.05 M and *I* = 0.30 M were prepared by dissolving varying amounts of MeCl (Me = Li, Na, K, Rb, Cs) in 0.005 M Me_2_CO_3_ solution of the corresponding cation. No buffer was used, and pH values varied between pH 10.3 and pH 10.9. Samples for the CE-ICP-MS measurements were prepared as described above. In addition to the U­(VI) stock solution ([^238^ *U*] = 2 × 10^–7^ M), an aliquot of 1 × 10^–5^ M HI was added to monitor the influence of ionic strength on the mobility of I^–^.

### 2.5 Influence of Buffers

To stabilize the pH values between pH 4 and pH 10, the “Good’s buffers”^22^ MES, HEPES, and CHES were used in this work. Although it is assumed that Good’s buffers show only weak affinity toward metal ions,^23,24^ data for actinide complexation is sparse at best. It should not be neglected that the buffers could complex actinides via their sulfonate group when discussing complex formation constants. Mandal et al.^25^ reported an interaction of several buffers with Eu­(III) and even proposed complex formation constants for Eu­(MES), Eu­(MES)_2_, and Eu­(HEPES) complexes. To check the extend of influence of MES and HEPES on the measurement, complex formation constants proposed by Mandal et al.^25^ for Eu­(III) as chemical analog for Am­(III) were added to the speciation calculation under the experimental conditions present in this work (Figure S1, Supporting Information). In the presence of MES, the Am­(MES)^2+^ and Am­(MES)_2_^+^ complexes form below 16% and thus hardly influence the trend in mobility (Figure S2, Supporting Information). The Am­(HEPES)^2+^ complex is not to be expected in the presence of carbonate. Therefore, the overall influence on the trend in electrophoretic mobility can be neglected. Th­(IV) is expected to show the highest affinity for the buffer. In absence of other ligands, an influence of CHES on the mobility of Th­(IV) at pH 10 was observed (not shown here). The mobilities measured in the presence of carbonate on the other hand were highly negative for the pH regions where the buffers were applied. Thus, Th­(IV)-buffer complexation in the presence of carbonate seems unlikely.

The influence of buffers on Np­(V) in the presence of carbonate has been sufficiently discussed by Topin et al.^14^ In the pH range where a U­(VI)-MES complex would be expected, the carbonate complexation of U­(VI) is already well advanced. No influence was observed for HEPES and CHES. It can be summarized that the influence of the buffers used can be neglected for the determination of actinide carbonate complex formation constants.

### 2.6 Data Treatment

For the determination of complex formation constants, the measured electrophoretic mobilities μ_eff_ were plotted against the pH value. By fitting eqs 12, S1, and S2 to the experimental data, complex formation constants log β were obtained, respectively. The fit was performed by a custom Python script using the lm-fit^26^ library based on the Levenberg–Marquardt method for nonlinear least-squares minimization. The corresponding correlation matrices are summarized in the SI. To simplify the fitting procedure, the mobilities μ_*i*_ of each species were fixed and varied separately. For the determination of μ_*i*_, the following conditions were applied:

(1) Positively charged complexes have positive mobilities and negatively charged complexes have negative mobilities.
(2) For neutral complexes, the electrophoretic mobility is fixed at zero.
(3) Electrophoretic mobilities decrease with the number of carbonate ligands associated with the actinide.
(4) If the trend in electrophoretic mobility shows a plateau at high [CO_3_^2–^] corresponding to the limiting complex, the value of the plateau is assumed to represent the mobility of the corresponding predominant complex and is fixed.

If not stated otherwise, all obtained complex formation constants were extrapolated to zero ionic strength using SIT.^21^ For this purpose, the ionic strength of *I* = 0.33 M was converted to molality units *I* = 0.333 mol/kg_w_ using the density of the NaCl solution calculated as described in Novotny and Sohnel.^27^ The other electrolytes were treated analogously. The ion interaction coefficients used in this work are summarized in SI (Table S6).

## 3 Results and Discussion

### 3.1 Carbonate Complexation

All electropherograms measured in NaCl can be found in SI (Figures S11 and S12). With eq 1, the effective electrophoretic mobilities μ_eff_ were calculated for each actinide (Table S8, Supporting Information). The electrophoretic mobilities μ_eff_ of Am­(III), Np­(V), and U­(VI) were plotted against the pH as shown in Figure 1.

**Figure 1:**
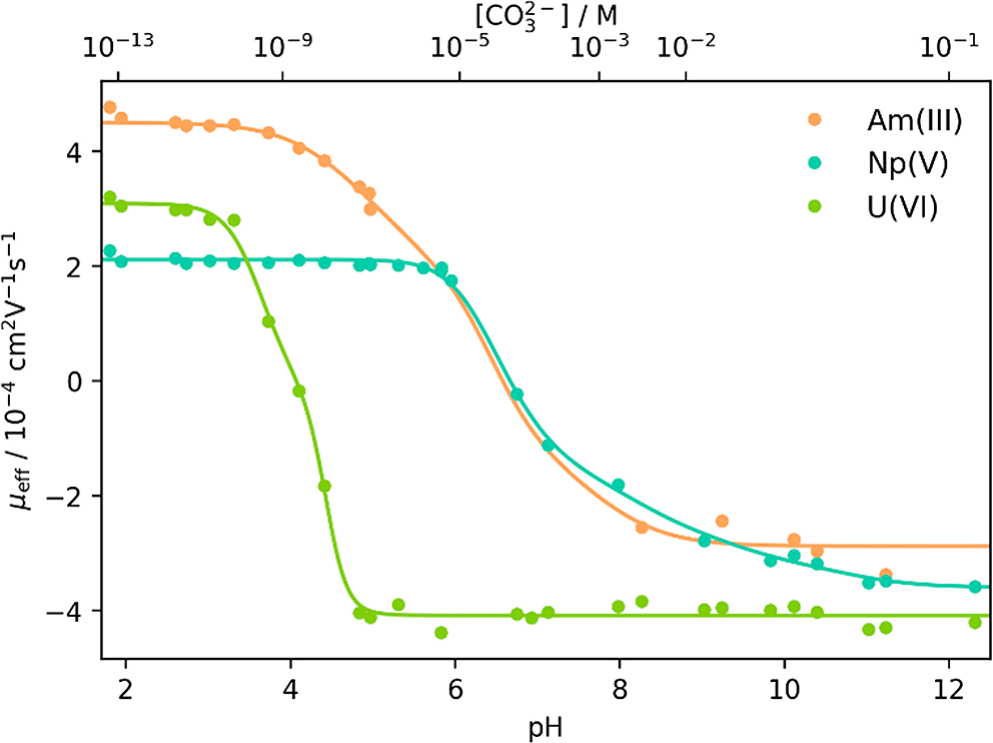
Measured effective electrophoretic mobilities μ_eff_ of ^241^Am(III), ^237^Np­(V), and ^238^U­(VI) plotted against pH as well as the free carbonate concentration [CO_3_^2–^] at *I* = 0.333 m in NaCl with applied fit (solid lines) according to eqs 12, S1, and S2. *R*_Am_^2^ = 0.997, *R*_Np_^2^ = 0.998, and *R*_U_^2^ = 0.998.

Clear progressions of μ_eff_ as a function of pH and carbonate concentration were observed for all actinides. The individual mobilities assigned to the actinide carbonate complexes are summarized in Table 2. Because of actinide chloride complexes, the electrophoretic mobility μ_0_ in absence of carbonate is made up of the mobility of the free actinide and the mobility of the chloride complex. The average charge *z* of (An^*z*+^ + AnCl^*z*–1^) given in Table 2 was calculated based on the proportions determined using PhreeQC^28^ and ThermoChimie v12a.^29^

**Table 2:** Assumed Electrophoretic Mobilities μ of the Individual Species Investigated^a^

species	*z*	μ/10^–4^ cm^2^/(Vs)	*Q*/10^4^ Vs/cm^2^
Am^3+^ + AmCl^2+^	2.93	4.50	0.65
Am(HCO_3_)^2+^	2	3.00	0.67
Am(CO_3_)^+^	1	1.53	0.65
Am(CO_3_)_2_^–^	–1	–1.53	0.65
Am(CO_3_)_3_^3–^	–3	–2.90	1.03
NpO_2_^+^	1	2.11	0.47
NpO_2_(CO_3_)^−^	–1	–1.64	0.61
NpO_2_(CO_3_)_2_^3–^	–3	–2.72	1.10
NpO_2_(CO_3_)_2_OH^4–^	–4	–3.59	1.11
NpO_2_(CO_3_)_3_^5–^	–5	–3.90	1.28
UO_2_^2+^ + UO_2_Cl^+^	1.90	3.09	0.61
UO_2_(CO_3_)_(aq)_	0	0	-
UO_2_(CO_3_)_2_^2–^	–2	–3.28	0.61
UO_2_(CO_3_)_3_^4–^	–4	–4.09	0.98

a The ionic charge of the species is *z*. For *Q* see eq 16 below.

#### 3.1.1 Americium­(III)

The trend in electrophoretic mobility (Figure 1) for Am­(III) starts and ends in a plateau. The corresponding values were selected as μ_0_ at low carbonate concentration and μ_3_ of the limiting 1:3 complex at high carbonate concentration. Values for the assumed mobilities of all actinide complexes investigated are summarized in Table 2. The mobility of Am­(CO_3_)_3_^3–^ agrees with the mobility of Eu­(III) measured by Philippini et al.^20^ using CE-ICP-MS at [Na_2_CO_3_] = 0.15 M. Between pH 5 and 8 electropherograms of Am did not show reliable peaks. This range of pH coincides with the pH range Chung et al.^30^ observed the formation of Cm­(III)-SiO surface complexes in 0.5 g/L amorphous silica solution. Therefore, a strong interaction or sorption of Am­(III) on the fused silica capillary is likely responsible for the loss of signal. In this pH range the Am­(CO_3_)_2_^–^ complex is expected to dominate. The lack of data points made it impossible to determine the complex formation constant of the 1:2 complex. For the fit of the data, the corresponding constant was expressed as log β_2_^0.333m^ *=* log β_1_^0.333m^ + log *K*_2_^0.333m^ with log *K*_2_^0.333m^ = 4.29, the value selected by Guillaumont et al.,^3^ extrapolated to *I* = 0.333 m, and fixed. At pH > 8 clear peaks for Am were observed, indicating that the formation of the 1:3 Am-CO_3_ complex reduces the sorption on the fused silica. The calculated complex formation constants log β_*i*_ are summarized in Table 3.

**Table 3:** Complex Formation Constants log β_*i*_ for Am­(III)-Carbonate Complexes at *I* = 0.333 m and Zero Ionic Strength as well as Values Selected in the NEA Review Done by Guillaumont et al.^3^

reaction	log β^0.333m^	log β_SIT_^0^	log β_NEA_^0^ ^3^
${\mathrm{Am}}^{3+}+CO_{3}^{2-}+H^{+}⇌\mathrm{Am}{\left(\mathrm{HC}O_{3}\right)}^{2+}$	12.20 ± 0.20	13.74 ± 0.20	13.43 ± 0.30^a^
$Am^{3+}+CO_{3}^{2-}⇌\mathrm{Am}{\left(CO_{3}\right)}^{+}$	6.69 ± 0.23	8.53 ± 0.23	8.00 ± 0.40
$Am^{3+}+2CO_{3}^{2-}⇌\mathrm{Am}(CO_{3}{)}_{2}^{-}$	10.98^b^	13.43	12.90 ± 0.60
$Am^{3+}+3CO_{3}^{2-}⇌\mathrm{Am}(CO_{3}{)}_{3}^{3-}$	13.84 ± 0.36	15.66 ± 0.36	15.00 ± 0.50^c^

a Calculated from selected data in Guillaumont et al.^3^ to match eq 5.

b Fixed at log β_2_^0.333m^ = log β_1_^0.333m^ + log * K*_2_^0.333m^ with log *K*_2_^0.333m^ = 4.29 extrapolated to *I* = 0.333 m from the value selected by Guillaumont et al.^3^ and fixed.

c The value was retained by the latest NEA review done by Grenthe et al.^5^ but the uncertainty was decreased.

The calculated log β^0^ value for the Am­(HCO_3_)^2+^ complex agrees with the value for the Cm­(HCO_3_)^2+^ complex selected in the NEA review done by Guillaumont et al.^3^ based on the experiments by Fanghänel et al.^9^ The obtained log β^0^ values for the Am­(CO_3_)^+^ and Am­(CO_3_)_3_^3–^ complexes also agree with the ones selected by Guillaumont et al.^3^ within the margin of error. To further compare the obtained values with the literature, the specific interaction equation^21^ was applied to the data compiled by Guillaumont et al.^3^ including the values determined in this work in Figure S3 in the SI. The inclusion of the values determined in this work does not change the values selected by Guillaumont et al.^3^ significantly.

#### 3.1.2 Neptunium­(V)

The measured electrophoretic mobilities for Np­(V) are in good agreement with the CE-ICP-MS study by Topin et al.^14^ under similar conditions (*I* = 0.37 M). The mobilities of Np­(V) are constant up to pH 5.5 (Figure 1). The value of this plateau was assigned to μ_0_ of NpO_2_^+^. At pH > 11 the electrophoretic mobility reaches plateau. The corresponding mobility was assigned to the NpO_2_(CO_3_)_2_OH^4–^ complex. The NpO_2_(CO_3_)_3_^5‑^complex only forms to a proportion <30%. This made an assignment of a mobility μ_3_ to the 1:3 complex difficult. The value for μ_3_ was determined as described by Topin et al.^14^ (see Table 2). In the preliminary fits, the free parameter β_3_ was constrained within the associated uncertainties of the value proposed by the NEA^2^ The calculated complex formation constants log β_*i*_ for the Np­(V) complexes are summarized in Table 4.

**Table 4:** Complex Formation Constants log β_*i*_ for Np­(V)-Carbonate Complexes at *I* = 0.333 m and Zero Ionic Strength as well as Values Selected in the NEA Reviews Done by Lemire et al.^2^ and Guillaumont et al.^3^ and Reported by Topin et al.^14^ and Aupiais et al.^15^

reaction	log β^0.333m^	log β_SIT_^0^	log β_NEA_^0^ ^2,3^	log β_Topin_^0^ ^14^	log β_Aupiais_^0^ ^15^
$\mathrm{Np}O_{2}^{+}\pm CO_{3}^{2-}⇌\mathrm{Np}O_{2}{\left(CO_{3}\right)}^{-}$	4.24 ± 0.04	4.81 ± 0.04	4.96 ± 0.06	4.88 ± 0.12	4.94 ± 0.08
$\mathrm{Np}O_{2}^{+}+2CO_{3}^{2-}⇌\mathrm{Np}O_{2}(CO_{3}{)}_{2}^{3-}$	6.68 ± 0.14	6.59 ± 0.14	6.53 ± 0.10	6.56 ± 0.10	6.45 ± 0.21
${\mathrm{NpO}}_{2}^{+}+3CO_{3}^{2-}⇌\mathrm{Np}O_{2}(CO_{3}{)}_{3}^{5-}$	7.51 ± 0.23	5.49 ± 0.23	5.50 ± 0.11	5.64 ± 0.15	5.54 ± 0.11
${\mathrm{NpO}}_{2}^{+}+2CO_{3}^{2-}+H_{2}O⇌\mathrm{Np}O_{2}(CO_{3}{)}_{2}OH^{4-}+H^{+}$	–3.88 ± 0.42	–5.20 ± 0.42	–5.31 ± 1.17^a^	-	-

a Calculated from selected data in Guillaumont et al.^3^ to match eq 6.

The calculated complex formation constants of the binary Np­(V)-carbonate complexes agree with those of the NEA^2^ as well as of Topin et al.^14^ and Aupiais et al.,^15^ showing the reproducibility of CE-ICP-MS when determining complex formation constants. Due to the agreement with data selected by Lemire et al.,^2^ applying the NEA procedure to determine the log β^0^ was not necessary. The NpO_2_(CO_3_)_2_OH^4–^complex was not determined by Topin et al.^14^ and Aupiais et al.^15^ The complex formation constant determined in this works agrees closely with the value selected by Lemire et al.^2^

#### 3.1.3 Uranium

The trend in electrophoretic mobility for U­(VI) starts and ends in a plateau (Figure 1). The corresponding μ_eff_ values were selected as μ_0_ at low carbonate concentration and μ_3_ of the limiting 1:3 complex at high carbonate concentration, respectively (Table 2).

The complex formation constants for U­(VI) deviate from literature^3^ (Table 5). The successive formation constants *K*_1_^0^ and *K*_2_^0^ deviate about one order of magnitude, while *K*_3_^0^ deviates two orders of magnitude. According to the present work, the U­(VI) carbonate complexation is stronger than previously assumed. The U­(VI) carbonate system has been studied extensively in literature through solubility experiments^16–18^ with the lowest U­(VI) concentrations around 3 × 10^–5^ M. At these concentrations, polynuclear U­(VI) complexes have a great influence on the speciation.^31^ At the U­(VI) concentration used in this work, polynuclear complexes are unlikely. Solubility experiments are also highly dependent on the characterization of solid phase,^5^ whereas with CE-ICP-MS complex formation can be studied directly in the liquid phase.

**Table 5:** Complex Formation Constants log β_*i*_ for U­(VI)-Carbonate Complexes at *I* = 0.333 m and Zero Ionic Strength as well as Values Selected in the NEA Review Done by Guillaumont et al.^3^

reaction	log β^0.333m^	log β_SIT_^0^	log β_NEA_^0^ ^3^
$UO_{2}^{2+}+CO_{3}^{2-}⇌UO_{2}(CO_{3}{)}_{\left(\mathrm{aq}\right)}$	9.52 ± 0.08	10.74 ± 0.08	9.94 ± 0.03
$UO_{2}^{2+}+2CO_{3}^{2-}⇌UO_{2}(CO_{3}{)}_{2}^{2-}$	17.13 ± 0.27	18.37 ± 0.27	16.61 ± 0.09
${\mathrm{UO}}_{2}^{2+}+3CO_{3}^{2-}⇌UO_{2}(CO_{3}{)}_{3}^{4-}$	25.30 ± 0.19	25.31 ± 0.19	21.84 ± 0.04

#### 3.1.4 Thorium

In contrast to the other actinides investigated, thorium only produced clear peaks at pH < 2.6. At pH values above 2.6, peaks in the electropherograms of ^232^Th started to show a lot of tailing or were interrupted (Figures S11 and S12, Supporting Information). Because of this, electrophoretic mobilities were difficult to determine. Samples were measured directly after Th­(IV) addition, remeasuring the samples produced worse signals with clear signs of precipitation and colloid formation^32^ (see Figure S4, Supporting Information). The significant amount of tailing indicates a retention of Th­(IV) on the fused silica of the capillary via surface complexation. In such cases the front of the signal was assumed to be only governed by electrophoretic mobility. For those reasons, the obtained electrophoretic mobilities are not sufficiently reliable for the determination of complex formation constants.

Nevertheless, a reproducible trend in Th­(IV) mobility was observed as shown by the data points in Figure 2. To verify this trend in μ_eff_, the electrophoretic mobility was modeled using the complex formation constants proposed in the NEA review by Rand et al.^4^ extrapolated to *I* = 0.333 m (see Figure 2 dashed line). Th­(IV) has a strong tendency to hydrolyze and to form ternary complexes which can be generalized by eq 14.

14 $${\mathrm{Th}}^{4+}+yOH^{-}+iCO_{3}^{2-}⇄\mathrm{Th}(\mathrm{OH}{)}_{y}(CO_{3}{)}_{i}^{4-y-2i}$$

**Figure 2:**
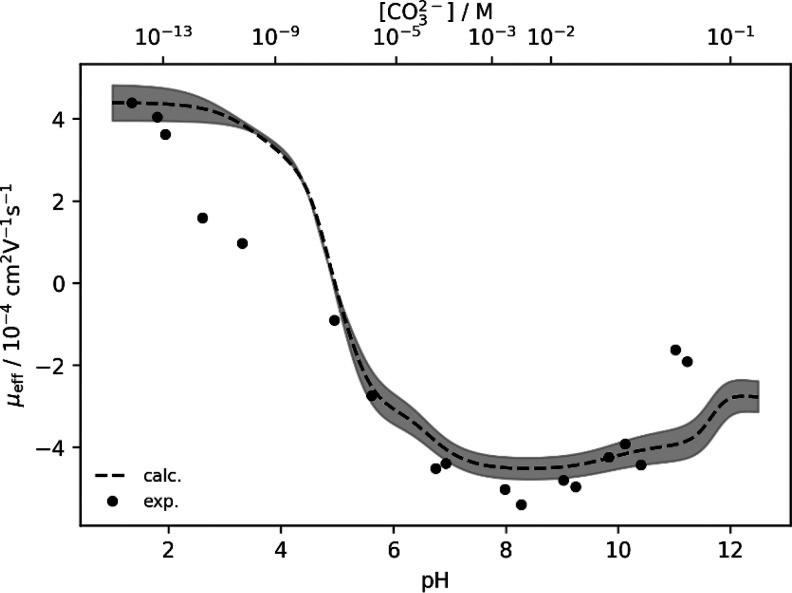
Measured effective electrophoretic mobilities μ_eff_ of ^232^Th(IV) (dots) plotted against pH value and [CO_3_^2–^] concentration. For comparison, μ_eff_ was estimated based on eq 15 using β_*i*_ values selected in the NEA review by Rand et al.^4^ (dashed line). The shaded area represents the uncertainty of log β values and a 90% confidence interval for the estimated mobilities.

Equation 12 was extended accordingly to account for the ternary complexes (eq 15).

15 $$\mu_{\mathrm{eff}}=\frac{\mu_{0}+\sum_{y,i}\mu_{y,i}\beta_{y,i}{{\left[OH^{-}\right]}^{y}\left[CO_{3}^{2-}\right]}^{i}}{1+\sum_{y,i}\beta_{y,i}{{\left[OH^{-}\right]}^{y}\left[CO_{3}^{2-}\right]}^{i}}$$

The hydroxide concentration was calculated using [OH^–^] = 10^–(p*K*_W_^0.333m^–pH)^ with p*K*_W_ = 13.73 at 0.333 m NaCl.

It is not feasible to include all possible binary and ternary Th­(IV) complexes to model the experimental data. The most important complexes were identified from speciation calculations using PhreeQC^28^ and ThermoChimie v12a^29^ (Figure S5, Supporting Information) and are listed in Table S1, Supporting Information. Unlike the other investigated actinides, the Th­(IV) mobility does not show a plateau at low carbonate concentrations, so μ_0_ = 4.39 × 10^–4^ cm^2^/(Vs) was estimated based on the measurement at pH 1.34, where no hydrolysis of the Th­(IV) is expected. For the Th–OH complexes, the mobilities were estimated based on the μ_0_ value and the change in ionic charge. The mobilities of the binary and ternary Th­(IV)–CO_3_ complexes were assigned based on the mobilities of other actinide species with the same charge (see Table 2). For the Th­(CO_3_)_5_^6–^ complex, there is no equivalently charged complex of the other actinides, so its mobility was estimated to be −5.4 × 10^–4^ cm^2^/(Vs).

As can be seen from Figure 2, a sharp decrease in the measured electrophoretic mobility in the pH range 2–3 was observed in deviation from the calculated values based on the NEA^4^ data. In this pH range the hydrolysis of Th­(IV) is negligible and a plateau in Th­(IV) mobility is expected. The observed decrease in mobility coincides with an increase in sorption of [Th] = 3 × 10^–5^ M on 4.8 g/L amorphous SiO_2_ in 1.0 M NaClO_4_ between pH 2 and 3 observed by Östhols.^33^ Therefore, the decrease in electrophoretic mobility at low pH could be attributed to the surface complexation of Th­(IV) on the fused silica of the capillary. The formation of Th–OH–CO_3_ complexes lead to a desorption of the Th. Between pH 5 and 10.5, the experimental data could be modeled well using literature data. Starting at pH 10, the mobility should become less negative due to the reduction in charge by formation of Th­(OH)_4_(CO_3_)^2–^ and Th­(OH)_3_(CO_3_)^−^. This reduction is seen in the experimental data, while the measured electrophoretic mobility around pH 11 deviates from the calculation either due to inaccurately assigned mobilities or a stronger contribution of the neutral Th­(OH)_4_ complex. Nevertheless, it was shown that the change in measured mobility can be explained to some extent with the literature values and the assumption of individual mobilities based on other actinide species.

To produce reliable measurements for the Th-OH-CO_3_ system, the experiments have shown that fused silica capillaries are not suitable. Recently, Sun et al.^34^ successfully utilized polyetheretherketone (PEEK) capillaries to reduce surface interaction while studying the Ca-U­(VI)-CO_3_ system.

#### 3.1.5 Electrophoretic Mobilities

Electrophoretic mobility is proportional to the charge-to-size ratio $\left(\frac{z}{r}\right)$ of the ions. To investigate the proportionality between the charge *z* and the mobility μ of a given species, to potentially see changes in ionic size *r*, a quotient *Q* is introduced as follows

16 $$Q=\frac{z}{\mu }$$

Most species investigated in this work have a *Q* value between 0.61 and 0.67 × 10^4^ Vs/cm^2^ (Table 2). Plotting the mobilities μ of the species investigated against the corresponding ionic charge *z* shows the linear progression more clearly (Figure 3).

**Figure 3:**
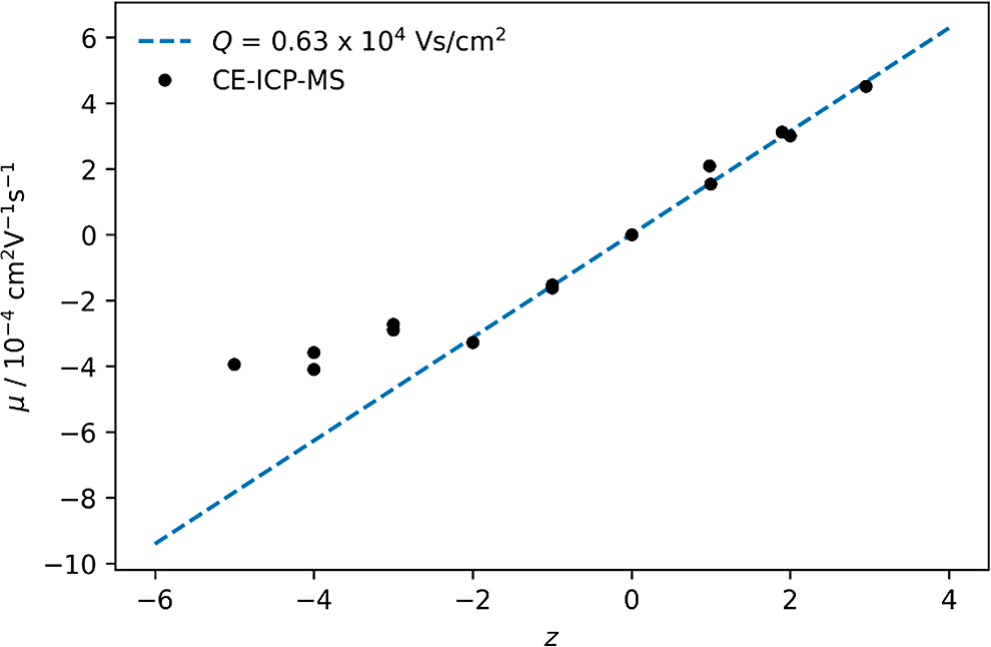
Individual mobilities μ of the species investigated in this work plotted against the corresponding ionic charge *z*. The blue line indicates a *Q* value of 0.63 × 10^4^ Vs/cm^2^.

For complexes with ionic charges between +3 and −2, the mobility follows a linear trend. A linear regression of the determined values in this range produces a *Q* value of (0.63 ± 0.01) × 10^4^ Vs/cm^2^. As *Q* is proportional to the ionic size *r*, it can be assumed that the association of carbonate ligands does not change the hydrodynamic radius of the complex species significantly as water molecules from the first hydration sphere are displaced.

For ionic charges below −2, the electrophoretic mobility deviates from the linear trend toward a less negative charge. This deviation increases with the negative charge of the complexes. This effect was also noticed by Aupiais et al.^15^ for CE-ICP-MS measurements of Pu­(V) in the carbonate system. Aupiais et al.^15^ attributed this change in mobility to a change in the binding motive from a bidentate coordination of the first two carbonate ligands to a monodentate coordination for the third ligand resulting in an increase of the coordination sphere. In the present study, the mobility of the NpO_2_(CO_3_)_2_^3–^ complex also shows a deviation from *Q* = 0.63 × 10^4^ Vs/cm^2^, although no change in the binding motif was observed for this complex by Aupiais et al.^15^ EXAFS studies showed a bidentate binding motive for all carbonate ligands in the limiting carbonate complexes of U­(IV),^35^ Np­(V),^36^ U­(VI),^37^ as well as in DFT studies for Am­(III).^38^ Without a change in binding motif, it is unlikely that the last carbonate ligand changes the hydrodynamic radius significantly. Another explanation, while also unlikely, could be the formation of ternary An-OH-CO_3_ complexes not described in previous literature and in turn a wrong assignment of anionic charge. As most of the formation constants determined in this work agree with previous literature, another effect might cause this deviation. Highly negative complexes are unlikely to be stable in aqueous solution and tend to reduce their negative charge by association with cations present in solution to form ternary alkali-actinide-carbonate complexes as proposed by Topin et al.^14^ For U­(VI),^39^ the association of 3.2 ± 0.7 Na^+^ ions to UO_2_(CO_3_)_3_^4–^ at a U–Na distance of 3.82 Å was observed using EXAFS. For U­(IV),^35^ EXAFS measurements in solution suggest the association of 2 or 3 Na^+^ ions to U­(CO_3_)_5_^6–^ at a U–Na distance of 3.62 Å. The formation of Na-UO_2_(CO_3_)_3_^4–^ ion pairs accompanied by a change in binding motif has also been proposed in theoretical calculations.^40^ To investigate this association further, the CE-ICP-MS experiments were extended to other alkali cations.

### 3.2 Influence of Alkali Cations

#### 3.2.1 Uranium

Due to the wide range of pH values as well as carbonate concentrations at which the UO_2_(CO_3_)_3_^4–^ complex is predominant, it is particularly suitable for further investigations into the effect of alkali cations. The mobility of ^238^U­(VI) was measured in 5 mM Me_2_CO_3_ solution at different ionic strengths using LiCl, NaCl, KCl, RbCl, and CsCl. All electropherograms can be found in SI (Figures S15 and S16, Supporting Information). With eq 1 the effective electrophoretic mobilities μ_eff_ were calculated for each electrolyte (Table S13, Supporting Information). The effective electrophoretic mobilities of U­(VI) are shown in Figure 4.

**Figure 4:**
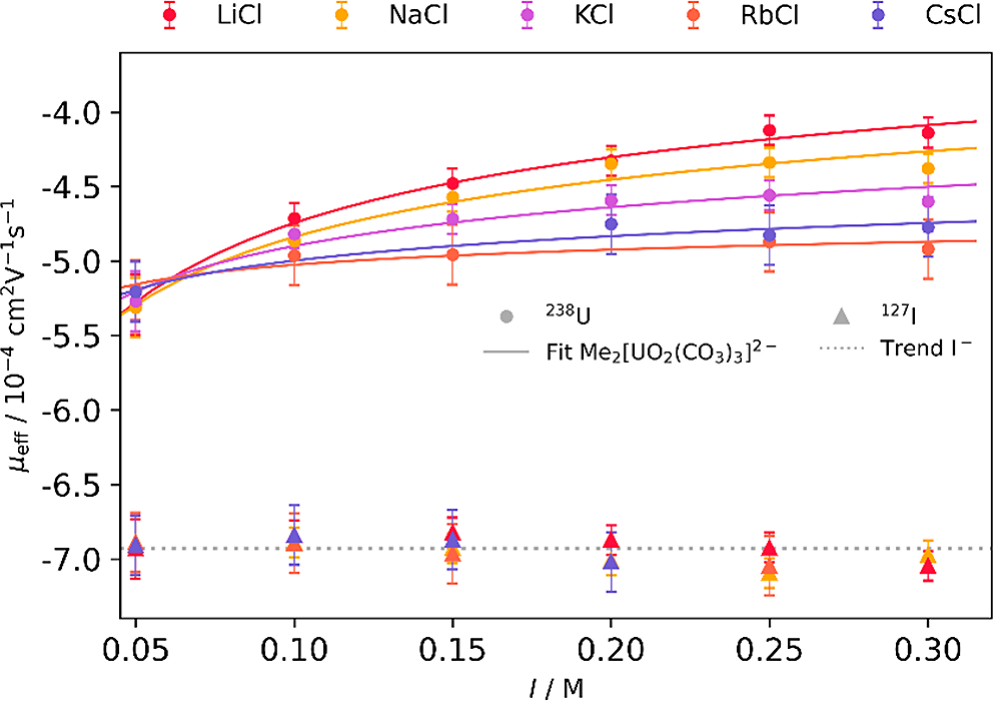
Measured effective electrophoretic mobilities μ_eff_ of ^127^ *I* and ^238^U­(VI) with [CO_3_^2‑^] = 5 mM in LiCl, NaCl, KCl, RbCl, and CsCl at pH between 10.3 and 10.9, plotted against the ionic strength with applied fit according to eq 19. *R*_Li_^2^ = 0.992, *R*_Na_^2^ = 0.964, *R*_K_^2^ = 0.865, *R*_Rb_^2^ = 0.868, and *R*_Cs_^2^ = 0.932.

The trend in mobility was found to be |μ_Li_| ≤ |μ_Na_| < |μ_K_| < |μ_Rb_| ≈ |μ_Cs_|. This trend was also observed by Philippini et al.^20^ for lanthanide carbonate complexes in different alkali chloride media (Li–K, Cs). For Li^+^, Na^+^, and K^+^ the effective mobility decreases significantly while for Rb^+^ and Cs^+^ the mobility only decreases slightly with increasing ionic strength or rather cation concentration. Iodide was added to the measurement to investigate the influence of the ionic strength on the electrophoretic mobility of negatively charged ions in general. It is expected that I^–^ does not form complexes with alkali ions under the experimental conditions. No apparent influence of the ionic strength or cation concentration on the mobility of I^–^ was noticed for the range investigated (Figure 4). Because of the higher anion charge of UO_2_(CO_3_)_3_^4–^ compared to I^–^, the ionic strength has a more significant influence on the activity of UO_2_(CO_3_)_3_^4–^ and in turn also possibly on the electrophoretic mobility. The formation of stable alkali complexes could superimpose the effect of ionic strength on mobility. For the following calculations, the effect of ionic strength on electrophoretic mobility in the investigated range is neglected. The change in electrophoretic mobility is only attributed to the formation of two ternary Me-U­(VI)-CO_3_ complexes based on eqs 17 and 18. These equilibria are described by the complex formation constant *K*_*x*_

17 $$K_{1}:,{\mathrm{Me}}^{+}+UO_{2}(CO_{3}{)}_{3}^{4-}⇌\mathrm{Me}{\mathrm{UO}}_{2}(CO_{3}{)}_{3}^{3-}$$

18 $$K_{2}:,{\mathrm{Me}}^{+}+\mathrm{MeU}O_{2}(CO_{3}{)}_{3}^{3-}⇌{\mathrm{Me}}_{2}{\mathrm{UO}}_{2}(CO_{3}{)}_{3}^{2-}$$

To calculate the complex formation constants *K*_*x*_ of the 1:1:3 and 2:1:3 Me_*x*_UO_2_(CO_3_)_3_^*x*–4^ complexes based on the mobilities shown in Figure 4, a function analogous to eq 12 was used.

19 $$\mu_{\mathrm{eff}}=\left\{\mu_{UO_{2}(CO_{3}{)}_{3}^{4-}}+\mu_{\mathrm{MeU}O_{2}(CO_{3}{)}_{3}^{3-}}\times K_{1}^{I}\times \left[Me^{+}\right]+\mu_{Me_{2}UO_{2}(CO_{3}{)}_{3}^{2-}}\times {K_{1}^{I}\times K}_{2}^{I}\times {\left[Me^{+}\right]}^{2}\right\}/\left\{1+K_{1}^{I}\times \left[Me^{+}\right]+{K_{1}^{I}\times K}_{2}^{I}\times {\left[Me^{+}\right]}^{2}\right\}$$

This equation is similar to that used by Sun et al.^34^ to investigate the complexation of Ca^2+^ with UO_2_(CO_3_)_3_^4–^. In contrast to Ca^2+^ in the experiments of Sun et al.,^34^ the alkali cations contribute predominantly to the ionic strength. Because of the varying ionic strength in the experiment, an extrapolation to zero ionic strength after the fit is not feasible. Instead, the complex formation constant at zero ionic strength *K* ^0^ is extrapolated to the ionic strength of each data point using SIT^21^ (eq 20). This way, the complex formation constants resulting from the fit are already extrapolated to zero ionic strength. The parameters Δ*z* ^2^ and Δε are summarized in Table 6.

20 $$\log K_{x}^{I}=\log K_{x}^{0}+\Delta z^{2}\times \left(\frac{0.509\sqrt{I_{m}}}{1+1.5\sqrt{I_{m}}}\right)-\Delta \varepsilon \times I_{m}$$

**Table 6:** Parameters Used for the Extrapolation of log *K* to a Given Ionic Strength

		1:1:3		2:1:3	
cation	ε (*j*, Cl^–^)	Δε	Δ*z* ^2^	Δε	Δ*z* ^2^
Li^+^	0.10	–0.10	–8	–0.11	–6
Na^+^	0.03	–0.03	–8	–0.04	–6
K^+^	0.00	0.00	–8	–0.01	–6
Rb^+^	0.00^a^	0.00	–8	–0.01	–6
Cs^+^	0.00^a^	0.00	–8	–0.01	–6

a ε­(*j*, Cl^–^) of Rb^+^ and Cs^+^ were estimated assuming ε­(*K* ^+^, Cl^–^) = ε­(Rb^+^/Cs^+^, Cl^–^).

The ion interaction parameters for the Me_*x*_UO_2_(CO_3_)_3_^*x*–4^ complexes are not known in literature. For Me_2_UO_2_(CO_3_)_3_^2–^, the ion interaction parameter of ε­(UO_2_(CO_3_)_2_^2–^, Na^+^) = −0.02 was used. As there is only a small change from ε­(UO_2_(CO_3_)_2_^2–^, Na^+^) = −0.02 to ε­(UO_2_(CO_3_)_3_^4–^, Na^+^) = −0.01, the latter value was used for the MeUO_2_(CO_3_)_3_^3–^ complex. It has to be noted that it is highly likely that the coefficients differ for the different cations. Since there is no literature, the coefficients of the U­(VI) complexes remained constant regardless of the cation for the evaluation. To simplify the evaluation, the molar ionic strength was equated with the molal ionic strength *I*_M_ = *I*_m_. This introduces a bias of at most 2% at 0.3 M CsCl and is therefore negligible.

Because of the carbonate in solution, the cation concentration deviated from the ionic strength as [Me^+^] = *I* – 0.005 M. The calculated complex formation constants *K*_x_^0^ are summarized in Table 7. The mobilities for the unassociated and associated complexes were calculated based on their ionic charge with eq 16 and a *Q* value of 0.63 × 10^4^ Vs/cm^2^ (see Table 8).

**Table 7:** Complex Formation Constants log *K*_x_^0^ for the Association of Alkali Cations to UO_2_(CO_3_)_3_^4–^ at Zero Ionic Strength

species	log *K*_Li_^0^	log *K*_Na_^0^	log *K*_K_^0^	log *K*_Rb_^0^	log *K*_Cs_^0^
MeUO_2_(CO_3_)_3_^3–^	1.07 ± 0.31	1.47 ± 0.28	2.00 ± 0.14	2.42 ± 0.09	2.25 ± 0.13
Me_2_UO_2_(CO_3_)_3_^2–^	2.50 ± 0.31	2.05 ± 0.27	1.44 ± 0.14	0.55 ± 0.21	0.97 ± 0.16

**Table 8:** Complex Formation Constants log β_*i*_ for U­(VI)-Carbonate Complexes and log *K*_*i*_ for Alkali U­(VI)-Carbonate Complexes at *I* = 0.33 M (Converted to Molality), as well as Estimated Electrophoretic Mobilities μ_*i*_ Based on a *Q* Value of 0.63 × 10^4^ Vs/cm^2^ ^a^

species	μ/10^–4^ cm^2^/(Vs)	log β_Li_^0.335m^	log β_Na_^0.333m^	log β_K_^0.332m^
UO_2_^+^ + UO_2_Cl^+^	3.09			
UO_2_(CO_3_)_(aq)_	0	9.74 ± 0.25	9.50 ± 0.12	9.89 ± 0.18
UO_2_(CO_3_)_2_^2–^	–3.17	17.70 ± 0.28	17.32 ± 0.22	17.77 ± 0.20
UO_2_(CO_3_)_3_^4–^	–6.35	24.23 ± 0.58	24.57 ± 0.25	24.12 ± 0.25

species	μ/10^–4^ cm^2^/(Vs)	log *K*_Li_^0.335m^	log *K*_Na_^0.333m^	log *K*_K_^0.332m^
MeUO_2_(CO_3_)_3_^3–^	–4.76	–0.16	0.22	0.74
Me_2_UO_2_(CO_3_)_3_^2–^	–3.17	1.42	1.34	1.24

a Adding the Me-U­(VI)-CO_3_ complexes to the fit enables the estimation of the mobilities of all complexes with a *Q* value of 0.63 × 10^4^ Vs/cm^2^.

Since the association of alkaline earth cations Mg^2+^–Ba^2+^ to UO_2_(CO_3_)_3_^4–^ was already investigated thoroughly in literature,^5^ the values selected by the NEA^5^ are listed in Table S2. In comparison, the log *K* ^0^ values for alkali metals are smaller than the log *K* ^0^ values for alkaline earth metals, which is plausible when considering the charge of the cations.^5^

For the MeUO_2_(CO_3_)_3_^3–^ complexes, log *K*_1_^0^ increases from Li^+^ to Rb^+^ and Cs^+^. The log *K*_2_^0^ values of Me_2_UO_2_(CO_3_)_3_^2–^ show an opposing unexpected trend with a decrease from Li^+^ to Rb^+^ and Cs^+^. For the latter two cations log *K*_1_^0^ as well as log *K*_2_^0^ are similar within the margin of error. This trend is further illustrated in the plot of log *K* ^0^ against the reciprocal ionic radius 1/*r* ^41^ (Figure S6, Supporting Information). The observed trends in log *K* ^0^ cannot easily be explained by trends in ionic or hydrodynamic radii. To fully understand the system, molecular dynamics calculations similar to the work of Li et al. for Na^+^ ^40^ are necessary for all investigated cations.

Overall, it must be noted that complex formation constants in the present work were calculated based on trends in electrophoretic mobilities, excluding further effects of ionic strength on said mobilities. The choice of electrophoretic mobility has an influence on the calculated complex formation constants. Nevertheless, the effect of the alkali cations on the UO_2_(CO_3_)_3_^4–^ complex was clearly demonstrated and represents a starting point for further investigations.

To check for consistency of the new complex formation constants (Table 7), they were fitted to the data of the experiment at varied pH values (Figure 1). In addition, this experiment was repeated analogously in LiCl/Li_2_CO_3_ and KCl/K_2_CO_3_ solutions. All electropherograms can be found in SI (Figures S13 and S14, Supporting Information). With eq 1 the effective electrophoretic mobilities μ_eff_ were calculated for each actinide (Tables S10, S12, Supporting Information). The measured effective electrophoretic mobilities of U­(VI) and the corresponding fits are shown in Figure 5.

**Figure 5:**
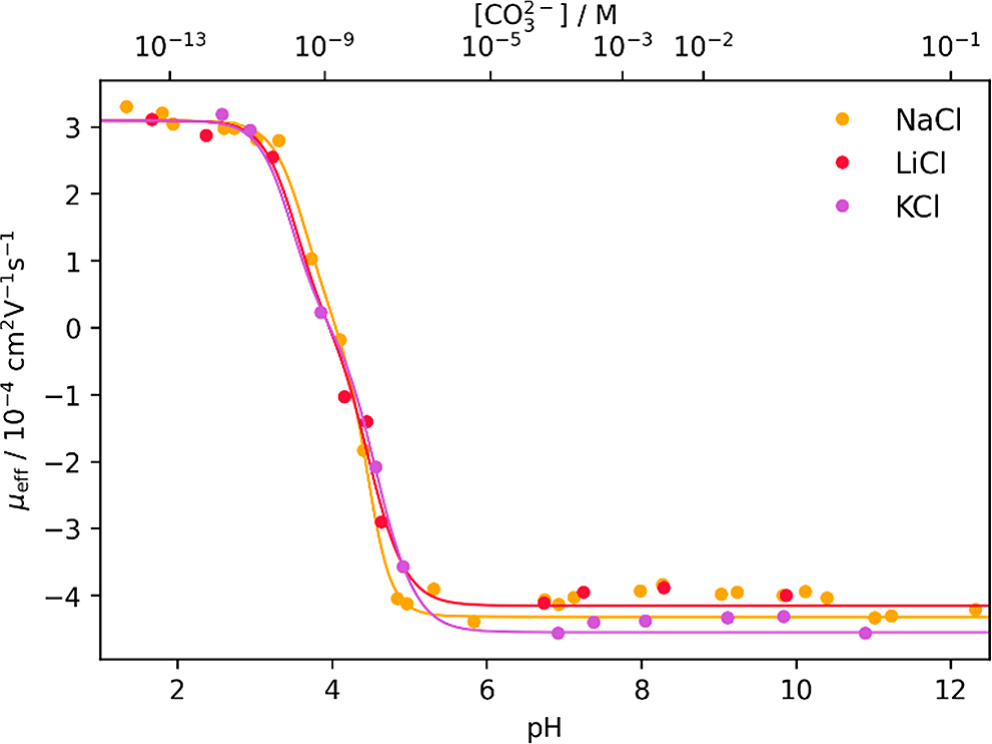
Measured effective electrophoretic mobilities μ_eff_ of ^238^U(VI) in LiCl, NaCl, and KCl plotted against the pH value and the free carbonate concentration [CO_3_^2–^] at *I* = 0.33 M with applied fit (solid lines) according to eq 21. *R*_Li_^2^ = 0.993, *R*_Na_^2^ = 0.996, and *R*_K_^2^ = 0.999.

The electrophoretic mobility μ_eff_ for U­(VI) in Figure 5 exhibits the same trend as observed in Figure 4 with an increase in mobility for the measurement in KCl at high [CO_3_^2–^] compared to the measurements in LiCl and NaCl.

To check the plausibility of the constants *K*_1_ and *K*_2_ (Table 7), the MeUO_2_(CO_3_)_3_^3–^ and Me_2_UO_2_(CO_3_)_3_^2–^ complexes were added to eq 12 to obtain eq 21.

21 $$\mu_{\mathrm{eff}}=\{\mu_{0}+\sum_{i}^{3}\mu_{i}\beta_{i}{\left[{\mathrm{CO}}_{3}^{2-}\right]}^{i}+{\mu_{1,3}\beta }_{3}K_{1}{\left[{\mathrm{CO}}_{3}^{2-}\right]}^{3}\left[{\mathrm{Me}}^{+}\right]+{\mu_{2,3}\beta }_{3}K_{1}K_{2}{\left[{\mathrm{CO}}_{3}^{2-}\right]}^{3}{\left[{\mathrm{Me}}^{+}\right]}^{2}\}/\{1+\sum_{i}^{3}\beta_{i}{\left[{\mathrm{CO}}_{3}^{2-}\right]}^{i}+\beta_{3}K_{1}{\left[{\mathrm{CO}}_{3}^{2-}\right]}^{3}\left[{\mathrm{Me}}^{+}\right]+\beta_{3}K_{1}K_{2}{\left[{\mathrm{CO}}_{3}^{2-}\right]}^{3}{\left[{\mathrm{Me}}^{+}\right]}^{2}\}$$

For the fits shown in Figure 5, all electrophoretic mobilities of the U­(VI) species, except for μ_0_, were calculated with a *Q* value of 0.63 × 10^4^ Vs/cm^2^ and were fixed during the fitting procedure (Table 8). The value for μ_0_ was again derived from the plateau at low [CO_3_^2–^] in NaCl (see Table 2). The complex formation constants log *K* ^0^ of the MeUO_2_(CO_3_)_3_^3–^ and Me_2_UO_2_(CO_3_)_3_^2–^ complexes in Table 7 were adjusted to *I* = 0.33 M (converted to molality) using SIT and fixed during the fitting procedure (Table 8). The concentration of the alkali cations was [Me^+^] = 0.3 M for the experiment. The electrophoretic mobilities, the adjusted log *K* ^*I*^ values, and the complex formation constants log β_*i*_ of the U­(VI)-CO_3_ complexes at *I* = 0.33 M (converted to molality) are summarized in Table 8.

Within the margin of error, the log β_*i*_ values for the 1:1 and 1:2 complexes in Table 8 coincide roughly with each other regardless of the alkali cation as well as with the log β_*i*_ values determined in Table 5 without the consideration of the ternary Me-U­(VI)-CO_3_ complexes. This shows that the alkali cation has next to no influence on the first two U­(VI)-CO_3_ complexes. For the complex UO_2_(CO_3_)_(aq)_, this is to be expected. Small differences in log β_*i*_ values compared to the values in Table 5 can be attributed to the slightly different mobility of the 1:2 complex.

For the 1:3 complex, the β_3_ value determined under consideration of the ternary Me-U­(VI)-CO_3_ complexes is about one order of magnitude smaller compared to the value determined without consideration of the ternary alkali complexes. Although for Na^+^ the β_3_ value is still about two orders of magnitude higher than the literature value, it is now closer to the literature,^5^ indicating an improvement of the model by including the alkali complexes.

Speciation diagrams for U­(VI) under the experimental parameters are shown in Figure S7 (Supporting Information) without and with consideration of the alkali complexes determined in this work. Speciation diagrams were calculated manually only utilizing the constants determined in this work (Tables 5 and 8).

By assuming the formation of alkali uranyl carbonate complexes, the limiting mobility of U­(VI) can be well modeled, which shows the consistency of the obtained complex formation constants over the different experiments.

#### 3.2.2 Neptunium

In addition to U­(VI), also Am­(III), Th­(IV), and Np­(V) were investigated in LiCl/Li_2_CO_3_ and KCl/K_2_CO_3_ solutions. For Am­(III) and Th­(IV), no satisfactory mobility curves could be obtained (not shown here). The trend in effective electrophoretic mobility μ_eff_ for Np­(V) in LiCl, NaCl, and KCl is shown in Figure 6. Measurements in LiCl reproduced the measurements in NaCl, while the mobilities in KCl deviate significantly at higher carbonate concentrations. In general, the mobilities follow the trend |μ_Li_| ≈ |μ_Na_| < |μ_K_| as observed for U­(VI).

**Figure 6:**
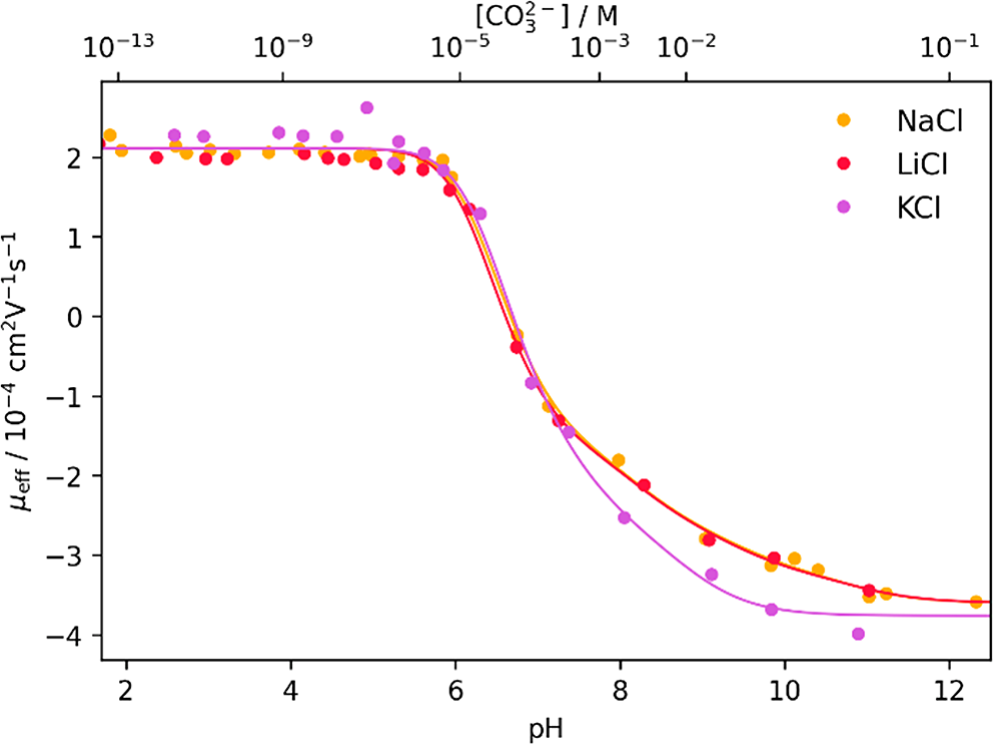
Measured effective electrophoretic mobilities μ_eff_ of ^237^Np(V) in LiCl, NaCl, and KCl plotted against the pH value and the free carbonate concentration [CO_3_^2–^] at *I* = 0.33 M with applied fit according to eq S2. *R*_Li_^2^ = 0.998, *R*_Na_^2^ = 0.998, and *R*_K_^2^ = 0.994.

In contrast to U­(VI), the carbonate complexation of Np­(V) does not reach the point where the limiting carbonate complex is almost 100% present in the investigated concentration range of carbonate. This complicates experiments to determine the alkali association, as at least two carbonate complexes are always present in significant proportions. The trend in electrophoretic mobility in LiCl and KCl was fitted analogously to the NaCl experiment by using the mobilities in Table 2. The complex formation constants log β_*i*_ for the three Np­(V) carbonate complexes at *I* = 0.33 M (converted to molality) are summarized in Table 9.

**Table 9:** Complex Formation Constants log β*_i_* for Np­(V)-Carbonate Complexes at *I* = 0.33 M LiCl, NaCl, and KCl (Converted to Molality), Calculated Using the Individual Mobilities of the Species in Table 2, Disregarding the Me-Np­(V)-CO_3_ Complexes

species	log β_Li_^0.335m^	log β_Na_^0.333m^	log β_K_^0.332m^
NpO_2_(CO_3_)^−^	4.31 ± 0.04	4.24 ± 0.04	4.21 ± 0.09
NpO_2_(CO_3_)_2_^3–^	6.77 ± 0.16	6.68 ± 0.14	7.30 ± 0.20
NpO_2_(CO_3_)_3_^5–^	7.65 ± 0.31	7.51 ± 0.23	9.18 ± 0.27
NpO_2_(CO_3_)_2_OH^4–^	–3.85 ± 0.74	–3.88 ± 0.42	-

The log β_*i*_ values for the 1:1 complex in Table 9 coincide with each other regardless of the alkali cation, indicating a negligible influence of the alkali cation. For the 1:2 and 1:3 complexes, the log β_*i*_ values for Li^+^ and Na^+^ also coincide with each other but show a significant deviation for K^+^. This suggests that the highly negatively charged Np­(V) carbonate or hydroxide carbonate complexes also form associated complexes with the alkali cations, analogous to U­(VI).

Because of this effect, ternary Me-Np­(V)-CO_3_ complexes were also included in the fit equation for the Np­(V) system (eqs S3 and S4, Supporting Information). To simplify the system, the range of carbonate concentration considered was limited to a maximum of 2 × 10^–2^ M. Thus, the influence of the 1:3 and 1:2:OH complexes can be neglected (see speciation Figure S8, Supporting Information). Analogous to U­(VI), MeNpO_2_(CO_3_)_2_^2–^ and Me_2_NpO_2_(CO_3_)_2_^–^ were considered as potential associated complexes. Because the experiment was conducted at a constant [Me^+^] of 0.3 M, it was not possible to produce a stable fit considering both complexes at the same time. Both stoichiometries were fitted separately based on the following equilibria

22 $$K_{1}:,{\mathrm{Me}}^{+}+\mathrm{Np}O_{2}(CO_{3}{)}_{2}^{3-}⇌\mathrm{Me}{\mathrm{NpO}}_{2}(CO_{3}{)}_{2}^{2-}$$

23 $$K_{2}:2{\mathrm{Me}}^{+}+\mathrm{Np}O_{2}(CO_{3}{)}_{2}^{3-}⇌{\mathrm{Me}}_{2}{\mathrm{NpO}}_{2}(CO_{3}{)}_{2}^{-}$$

Except for μ_0_, all electrophoretic mobilities of the Np­(V) species were calculated with a *Q* value of 0.63 × 10^–4^ Vs/cm^2^ and were fixed during the fitting procedure. The value for μ_0_ was again derived from the plateau at low [CO_3_^2–^] in NaCl. The results for each fit are shown in the SI (Tables S3 and S4, Figures S9 and S10, Supporting Information). The results from the most plausible fits are summarized in Table 10.

**Table 10:** Complex Formation Constants log β_*i*_ for Np­(V)-Carbonate Complexes and log *K*_*i*_ for Alkali Np­(V)-Carbonate Complexes at *I* = 0.33 M as well as Estimated Electrophoretic Mobilities μ_*i*_ Based on a *Q* Value of 0.63 × 10^4^ Vs/cm^2^, under the Consideration of Me-Np­(V)-CO_3_ Complexes

species	μ/10^–4^ cm^2^/(Vs)	log β_Li_^0.335m^	log β_Na_^0.333m^	logβ_K_^0.332m^
NpO_2_^+^ + NpO_2_Cl_(aq)_	2.11			
NpO_2_(CO_3_)^−^	–1.59	4.34 ± 0.05	4.25 ± 0.04	4.25 ± 0.09
NpO_2_(CO_3_)_2_^3–^	–4.76	6.25 ± 0.17	6.13 ± 0.15	6.17 ± 0.52

species	μ/10^–4^ cm^2^/(Vs)	log *K*_Li_^0.335m^	log *K*_Na_^0.333m^	log *K*_K_^0.333m^
MeNpO_2_(CO_3_)_2_^2–^	–3.17	-	-	1.40 ± 0.71
Me_2_NpO_2_(CO_3_)_2_^–^	–1.59	1.03 ± 0.19	1.01 ± 0.17	-

For K^+^, the addition of the MeNpO_2_(CO_3_)_2_^2–^ complex produces the most plausible log β_*i*_ values with both values for the 1:1 and 1:2 complexes agreeing with the literature^2^ within the margin of error. For Li^+^ and Na^+^, the addition of the MeNpO_2_(CO_3_)_2_^2–^ complex (Table S3, Supporting Information) underestimates the β_*i*_ value of the 1:2 complex by one to two orders of magnitude compared to literature.^2^ The Me_2_NpO_2_(CO_3_)_2_^–^ complex better describes the charge compensation that is observed for Li^+^ and Na^+^. By adding this complex for Li^+^ and Na^+^, the log β_*i*_ values for the 1:1 and 1:2 Np­(V)-CO_3_ complexes coincide with each other regardless of the alkali cation and are in range of the literature.^2^ The estimated complex formation constants for the Me_2_NpO_2_(CO_3_)_2_^–^ complex with Li^+^ and Na^+^ are identical. Note that the estimation only considers one associated complex and the preceding alkali complex was neglected. Thus, the log *K* value of the Me_2_NpO_2_(CO_3_)_2_^−^ complexes could be overestimated.

## 4 Conclusion

With the coupling between CE and ICP-MS, it was possible to determine formation constants for the carbonate complexes of Am­(III), Th­(IV), Np­(V), and U­(VI) simultaneously at low concentrations (μM). This enabled the investigation of only mononuclear actinide carbonate complexes. For Am­(III) and Np­(V), literature values could be reproduced for the most part. The carbonate complexation of U­(VI) was found to be stronger than previously reported in literature. It was not possible to measure Th­(IV) with enough certainty to determine complex formation constants, but the observed trend in electrophoretic mobility at pH > 5 was as expected based on literature data.

The sensitivity of the CE-ICP-MS to the charge of a complex allows investigations of association reactions, which would go unnoticed using other methods for speciation in liquid phases. For highly negatively charged actinide-carbonate complexes, a significant influence of alkali cations on the electrophoretic mobility was observed. This effect could be attributed to the association of cations to those complexes. The electrophoretic mobility of negatively charged An-CO_3_ complexes was found to follow the trend |μ_Li_| ≤ |μ_Na_| < |μ_K_|. Furthermore, the reaction of UO_2_(CO_3_)_3_^4–^ with different alkali cations (Li^+^–Cs^+^) was studied. The complex formation constants of MeUO_2_(CO_3_)_3_^3–^ and Me_2_UO_2_(CO_3_)_3_^2–^ for all investigated cations were determined. A similar influence of alkali cations (Li^+^–K^+^) on the carbonate complexation of Np­(V) was observed and complex formation constants of KNpO_2_(CO_3_)_2_^2–^ and Me_2_NpO_2_(CO_3_)_2_^–^ for Li^+^ and Na^+^ were estimated.

More work is needed to understand the role of cations in the electrolyte, which are no longer seen as innocent bystanders but rather seem to take part in the reaction. Even though CE-ICP-MS proved to be a powerful tool for investigating these cation interactions, more experiments and molecular dynamics calculations are needed to further investigate ternary alkali-actinide-carbonate complexes in solution.

## Supplementary Material



## Notes

Additional CE-ICP-MS data, speciation diagrams, experimental parameters, and electropherograms (PDF)
