# Supplementary material for: CE-ICP-MS Investigation of the Carbonate Complexation of Am(III), Th(IV), Np(V), and U(VI) and the Influence of Alkali Cations
Source: Inorg Chem. 2025 Oct 16;64(42):20915–26. doi: 10.1021/acs.inorgchem.5c02509 (PMC12570134; doi:10.1021/acs.inorgchem.5c02509)
Supplement: Supplementary file 1 [file ic5c02509_si_001.pdf]

SUPPORTING INFORMATION

CE-ICP-MS Investigation of the Carbonate Complexation of Am(III), Th(IV), Np(V), and U(VI) and the Influence of Alkali Cations

*Janik Lohmann[1], Stefanie Isabella Demel[1], Justus Carl Sander[1], and Tobias Reich[1]**

[1]Johannes Gutenberg-Universität Mainz, Department of Chemistry-Nuclear Chemistry, 55099 Mainz, Germany

* Corresponding author: treich@uni-mainz.de

The Supporting Information comprises 22 pages, including 16 figures, 13 tables, and 13 references.

Contents

Additional data 2

- **Americium** 2
- **Thorium** 3
- **Uranium** 5
- **Neptunium** 6

Chemicals 9

Ion Interaction Coefficients 10

Raw Data 11

Electropherograms 16

Correlation matrices 21

- **Figure 1-main** 21
- **Figure 4-main** 21

References 22

Additional data

Americium

$$\mu_{eff} = \frac{\mu_0 + \mu_{1,\mathrm{H}}\beta_{1,\mathrm{H}}[\mathrm{CO_3^{2-}}][\mathrm{H^+}] + \sum_{i=1}^{3}\mu_i\beta_i[\mathrm{CO_3^{2-}}]^i}{1 + \mu_{1,\mathrm{H}}\beta_{1,\mathrm{H}}[\mathrm{CO_3^{2-}}][\mathrm{H^+}] + \sum_{i=1}^{3}\beta_i[\mathrm{CO_3^{2-}}]^i} \quad \text{(S1)}$$

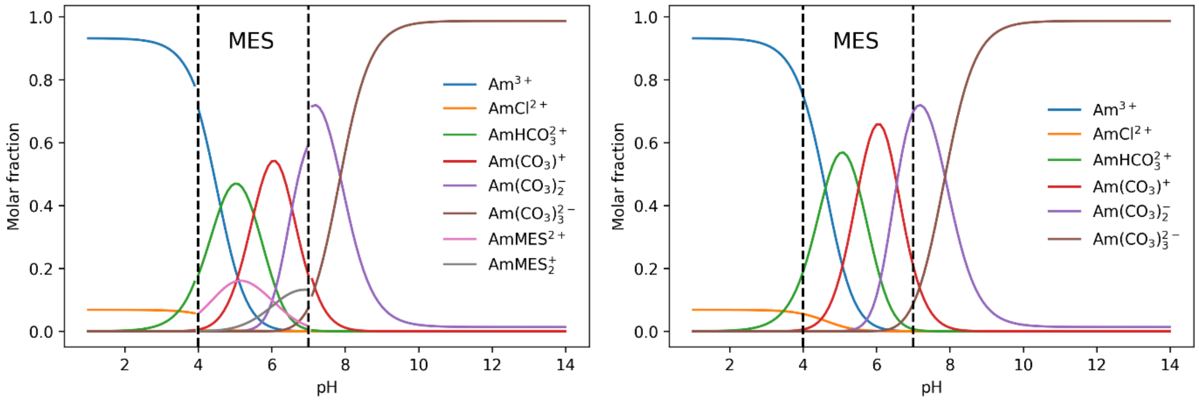

Figure S1. Speciation diagrams for Am(III) with (left) and without (right) Am(III)-MES complexes, calculated manually from the complex formation constants selected in the NEA review done by Guillaumont et al.[1] extrapolated to the ionic strength using SIT as well as Eu(III)-MES constants proposed by Mandal et al.[2] as an analog for Am(III)-MES extrapolated to the ionic strength using the Davies equation.[3], $[\mathrm{Me_2CO_3}] = 0.1$ M, $I = 0.333$ m (NaCl), [HMES] = 50 mM.

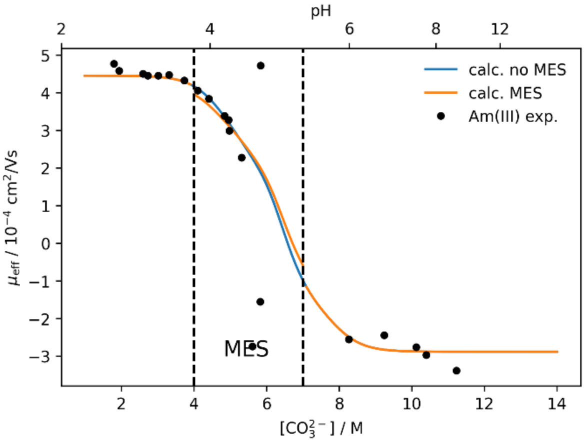

Figure S2. Comparison of the trends in effective electrophoretic mobility μ_{eff} calculated from Figure S1 using the individual electrophoretic mobilities from Table 2-main. The mobilities of $\mathrm{AmMES^{2+}}$ and $\mathrm{AmMES_2^{+}}$ were estimated at 3.08×10^{-4} and 1.53×10^{-4} cm²/Vs, respectively. The experimental data are shown as black circles. As can be seen, the formation of Am-MES complexes does not influence the trend in mobility significantly.

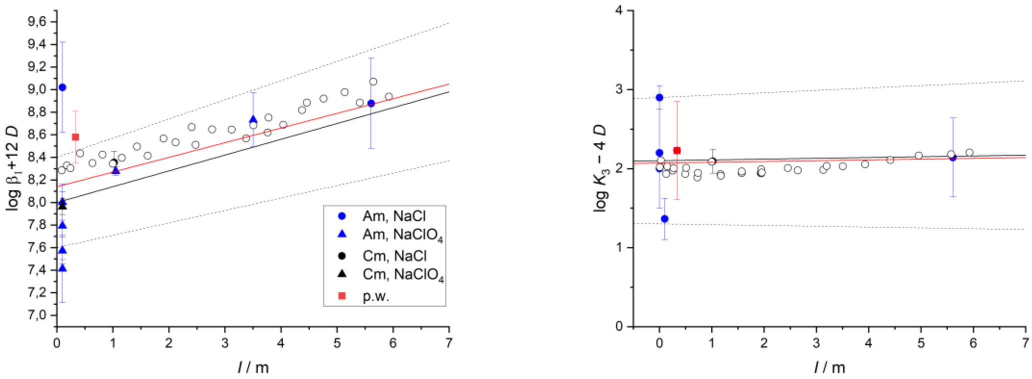

Figure S3. Extrapolation to I = 0 of experimental data for the formation of $\mathrm{Am(CO_3)^{+}}$ (left) and $\mathrm{Am(CO_3)_3^{3-}}$ (right) using the specific interaction equation[3]. The data were taken from the compilation in the NEA review done by Guillaumont et al.[1] For the fit, only data obtained for Am(III) in NaCl were utilized as well as studies performed at 0.1 M $NaClO_4$, as at this ionic strength only a negligible difference between NaCl and $NaClO_4$ is expected. Black solid lines show the fit based on the data selected by Guillaumont et al.[1], the dashed line represents the uncertainties and the red solid line show the fit performed including the value obtained in this work. The data for Cm(III) and higher $NaClO_4$ concentrations are only shown for comparison. The open circles show the data obtained by Fanghänel et al.[4] For the $\mathrm{Am(CO_3)^{+}}$ complex, $\log \beta_1^0 = 8.15 \pm 0.29$ and $\Delta\varepsilon = -(0.13 \pm 0.11)$ were obtained. The values do not differ significantly from $\log \beta_1^0 = 8.00 \pm 0.40$ and $\Delta\varepsilon = -(0.14 \pm 0.03)$ selected by Guillaumont et al.[1] For the $\mathrm{Am(CO_3)_3^{3-}}$ complex, $\log K_3^0 = 2.07 \pm 0.23$ and $\Delta\varepsilon = -(0.01 \pm 0.08)$ were obtained. The values again do not differ significantly from $\log K_3^0 = 2.1 \pm 0.8$ $\Delta\varepsilon = -(0.01 \pm 0.02)$ selected by Guillaumont et al.[1]

Thorium

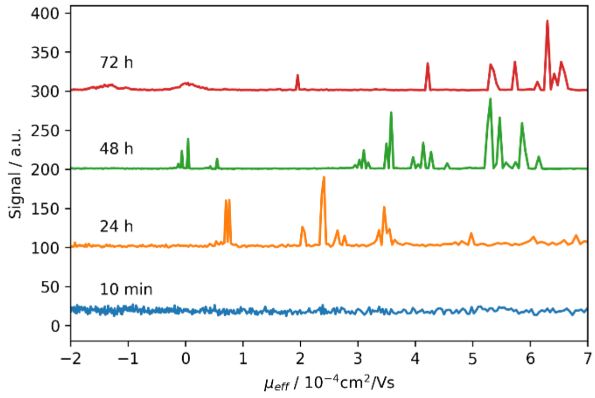

Figure S4. Electropherograms of ^{232}Th in 0.1 M Na_2CO_3 solution, I = 0.333 m (NaCl), pH = 4.84, normalized signal, l = 50 cm, measured at 10 kV over the course of four days. Directly after the addition of Th(IV), no signal was observed. After 24 h, sharp signals appeared caused by colloidal particles entering the plasma. The colloidal signal persisted in all further measurements.

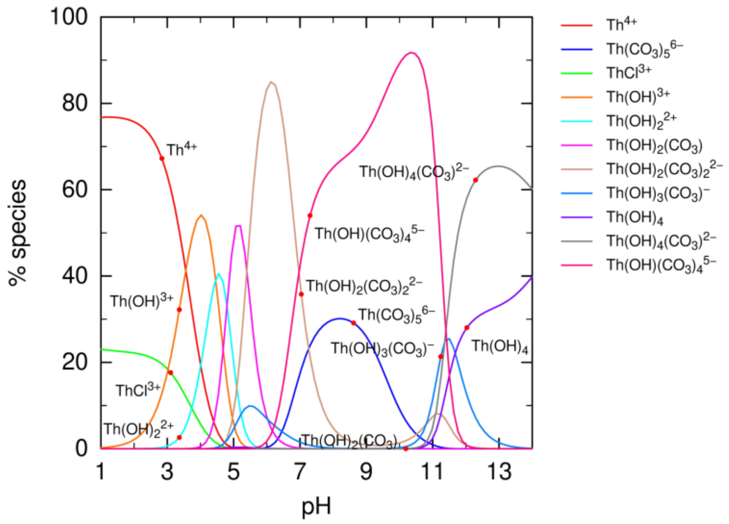

Figure S5. Speciation diagrams for Th(IV)-CO_3, calculated using PhreeQC[5] and ThermoChimie v12a[6] (SIT), plotted with PhreePlot[7] [Na_2CO_3] = 0.1 M, [Th] = 1×10^{-6} M, [NaCl] = 0.1 M, pH fixed with NaOH.

Table S1. Complex formation constants log β_i for $Th(OH)_y(CO_3)_i{}^{4-y-2i}$ complexes proposed in the NEA review by Rand et al.[9] extrapolated to I = 0.333 m as well as estimated electrophoretic mobilities based on the other actinides.

Reaction/Species	μ / 10^{-4} cm^2/(Vs)	log $\beta^{0.333\ m}$	log β^0_{NEA} [9]
Th^{4+} + $ThCl^{3+}$	4.39		
$Th^{4+} + OH^- \rightleftharpoons Th(OH)^{3+}$	3.3	10.3 ± 0.5	11.5 ± 0.5
$Th^{4+} + 2OH^- \rightleftharpoons Th(OH)_2^{2+}$	2.2	19.9 ± 0.5	21.8 ± 0.5
$Th^{4+} + 4OH^- \rightleftharpoons Th(OH)_{4\ (aq)}$	0	35.6 ± 0.5	38.6 ± 0.7
$Th^{4+} + 5CO_3^{2-} \rightleftharpoons Th(CO_3)_5^{6-}$	−5.4	31.0 ± 0.7	31.0 ± 0.7
$Th^{4+} + OH^- + 4CO_3^{2-} \rightleftharpoons Th(OH)(CO_3)_4^{5-}$	−3.90	34.4 ± 0.5	35.6 ± 0.5
$Th^{4+} + 2OH^- + CO_3^{2-} \rightleftharpoons Th(OH)_2(CO_3)_{(aq)}$	0	27.1 ± 0.6	30.5 ± 0.6
$Th^{4+} + 2OH^- + 2CO_3^{2-} \rightleftharpoons Th(OH)_2(CO_3)_2^{2-}$	−3.28	33.4 ± 0.5	36.8 ± 0.5
$Th^{4+} + 3OH^- + CO_3^{2-} \rightleftharpoons Th(OH)_3(CO_3)^-$	−1.53	34.9 ± 0.7	38.3 ± 0.7
$Th^{4+} + 4OH^- + CO_3^{2-} \rightleftharpoons Th(OH)_4(CO_3)^{2-}$	−3.28	37.4 ± 0.6	40.4 ± 0.6

Uranium

Table S2. Complex formation constants log K_x^0 for the association of alkaline earth cations to $UO_2(CO_3)_3^{4-}$ at I = 0 M selected by NEA[3].

**Species**	**log K_{Mg}^0**	**log K_{Ca}^0**	**log K_{Sr}^0**	**log K_{Ba}^0**
$MeUO_2(CO_3)_3^{2-}$	4.4 ± 0.2	5.2 ± 0.2	4.1 ± 0.2	3.8 ± 0.3
$Me_2UO_2(CO_3)_{3\,(aq)}$	0.9 ± 0.6	3.7 ± 0.4	3.8 ± 0.5	-

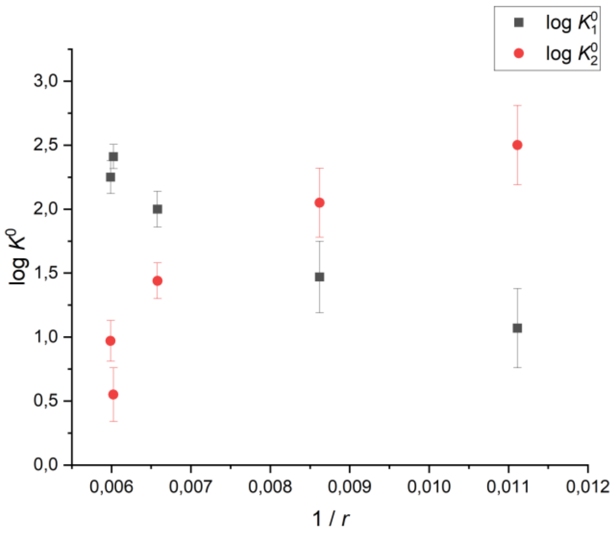

Figure S6. Plot of log K^0 for the formation of $MeUO_2(CO_3)_3^{3-}$ and $Me_2UO_2(CO_3)_3^{2-}$ against the reciprocal ionic radius 1/r of the different alkali cations.[10]

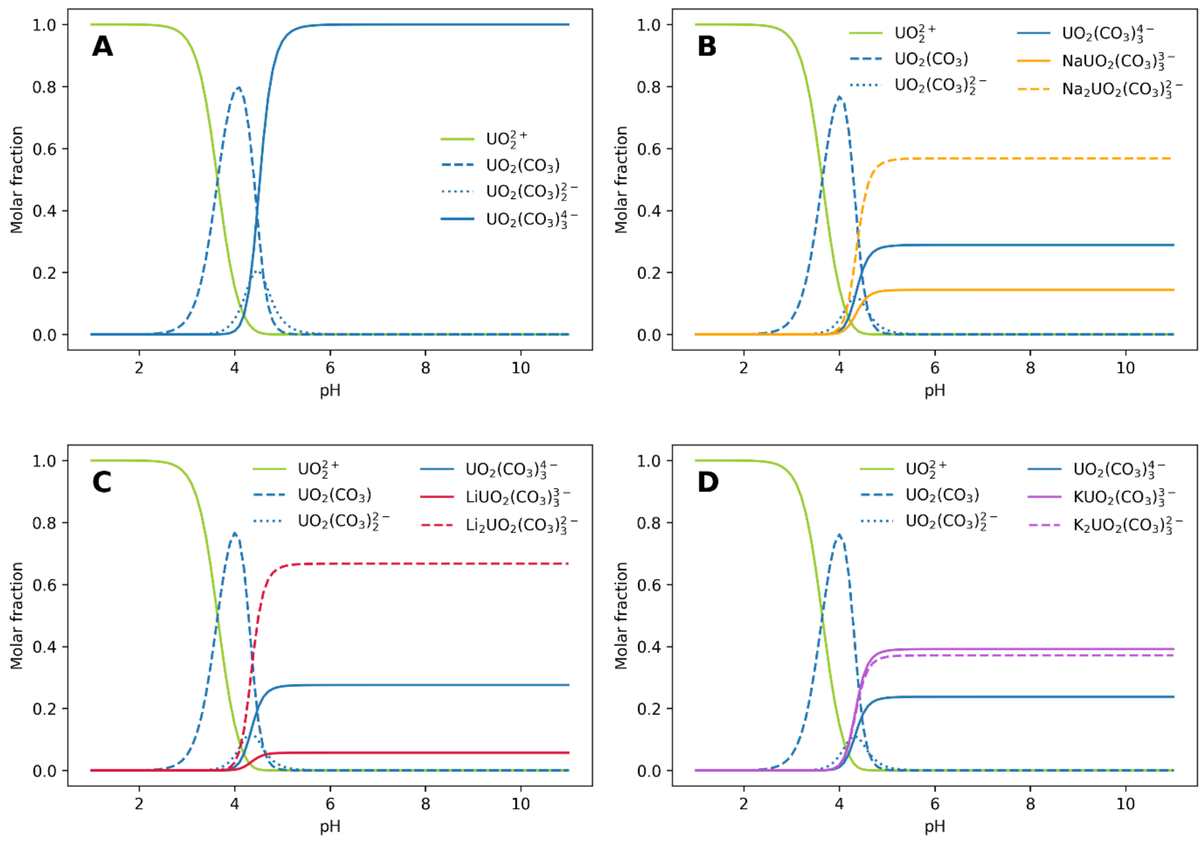

Figure S7. Speciation diagrams for U(VI) without (A) and with alkali complexes (B: Na^+, C: Li^+, D: K^+), calculated manually from the complex formation constants derived from the present work (Table 5-main, Table 8-main), UO_2Cl^+ is neglected, $[Me_2CO_3]$ = 0.1 M, I = 0.33 M, $[Me^+]$ = 0.3 M.

Neptunium

$$\mu_{eff} = \frac{\mu_0 + \mu_{1,\mathrm{OH}}\beta_{1,\mathrm{OH}}[\mathrm{CO_3^{2-}}][\mathrm{H^+}]^{-1} + \sum_{i=1}^{3}\mu_i\beta_i[\mathrm{CO_3^{2-}}]^i}{1 + \mu_{1,\mathrm{OH}}\beta_{1,\mathrm{OH}}[\mathrm{CO_3^{2-}}][\mathrm{H^+}]^{-1} + \sum_{i=1}^{3}\beta_i[\mathrm{CO_3^{2-}}]^i} \quad \text{(S2)}$$

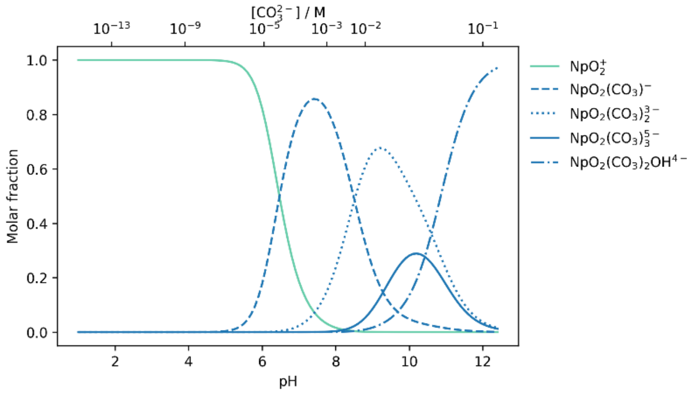

Figure S8. Speciation diagram for Np(V) without alkali complexes, calculated manually from the complex formation constants derived from the present work (Table 4-main), $[Na_2CO_3]$ = 0.1 M, I = 0.333 m.

Assumption 1: $MeNpO_2(CO_3)_2^{2-}$

$$\mu_{\text{eff}} = \frac{\mu_0 + \sum_i^2 \mu_i \beta_i [CO_3^{2-}]^i + \mu_{1,2}\beta_2 K_1 [CO_3^{2-}]^3[Me^+]}{1 + \sum_i^2 \beta_i [CO_3^{2-}]^i + \beta_2 K_1 [CO_3^{2-}]^3[Me^+]} \quad (S3)$$

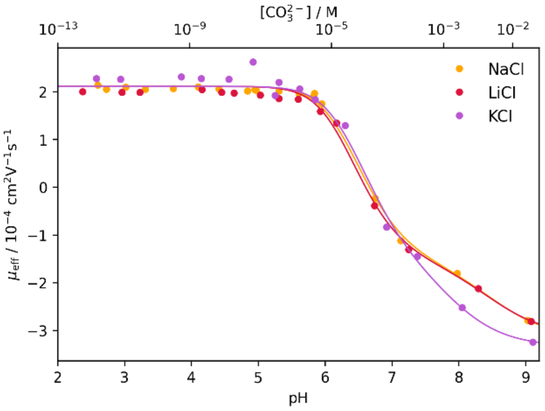

Figure S9. Measured effective electrophoretic mobilities of [237]Np(V) in LiCl, NaCl, and KCl plotted against the pH value as well as the free carbonate concentration free carbonate concentration $[CO_3^{2-}]$ at I = 0.33 M with applied fit according to eq. S3 with consideration of the $MeNpO_2(CO_3)_2^{2-}$ complex. R_{Li}^2 = 0.997, R_{Na}^2 = 0.996, and R_K^2 = 0.992.

Table S3. Complex formation constants log β_i for Np(V) carbonate complexes and log K for the $MeNpO_2(CO_3)_2^{2-}$ complex at I = 0.33 M as well as estimated electrophoretic mobilities μ based on a Q value of 0.63 × 10^4 Vs/cm^2.

Species	μ / 10^{-4} cm^2/(Vs)	log $\beta_{Li}^{0.335\text{ m}}$	log $\beta_{Na}^{0.333\text{ m}}$	log $\beta_{K}^{0.332\text{ m}}$
$NpO_2^+ + NpO_2Cl_{(aq)}$	2.11			
$NpO_2(CO_3)^-$	−1.59	4.34 ± 0.05	4.25 ± 0.04	4.25 ± 0.09
$NpO_2(CO_3)_2^{3-}$	−4.76	4.80 ± 5.02	5.08 ± 1.65	6.17 ± 0.52

Species	μ / 10^{-4} cm^2/(Vs)	log $K_{Li}^{0.335\text{ m}}$	log $K_{Na}^{0.333\text{ m}}$	log $K_{K}^{0.332\text{ m}}$
$MeNpO_2(CO_3)_2^{2-}$	−3.17	2.26 ± 5.03	1.83 ± 1.92	1.40 ± 0.71

Assumption 2: $Me_2NpO_2(CO_3)_2^-$

$$\mu_{\mathrm{eff}} = \frac{\mu_0 + \sum_i^2 \mu_i \beta_i [CO_3^{2-}]^i + \mu_{2,2}\beta_2 K_2 [CO_3^{2-}]^3 [Me^+]^2}{1 + \sum_i^2 \beta_i [CO_3^{2-}]^i + \beta_2 K_2 [CO_3^{2-}]^3 [Me^+]^2} \tag{S4}$$

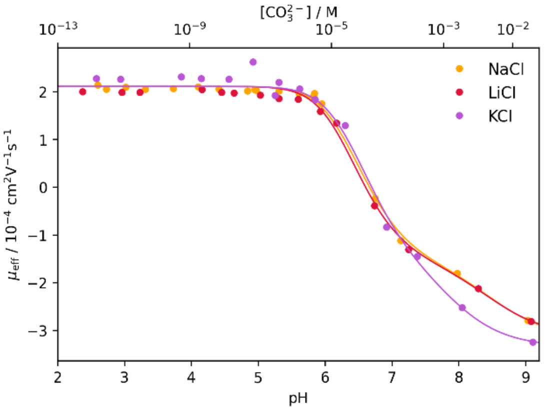

Figure S10. Measured effective electrophoretic mobilities of [237]Np(V) in LiCl, NaCl, and KCl plotted against the pH value as well as the free carbonate concentration free carbonate concentration $[CO_3^{2-}]$ at $I = 0.33$ M with applied fit according to eq. S4 with consideration of the $Me_2NpO_2(CO_3)_2^-$ complex. $R_{Li}^2 = 0.997$, $R_{Na}^2 = 0.996$, and $R_K^2 = 0.992$.

Table S4. Complex formation constants log β_i for Np(V) carbonate complexes and log K for the $Me_2NpO_2(CO_3)_2^-$ complex at $I = 0.33$ M as well as estimated electrophoretic mobilities μ based on a Q value of 0.63×10^4 Vs/cm^2.

Species	μ / 10^{-4} cm^2/(Vs)	log $\beta_{Li}^{0.335\ m}$	log $\beta_{Na}^{0.333\ m}$	log $\beta_{K}^{0.332\ m}$
$NpO_2^+ + NpO_2Cl_{(aq)}$	2.11			
$NpO_2(CO_3)^-$	−1.59	4.34 ± 0.05	4.25 ± 0.04	4.26 ± 0.09
$NpO_2(CO_3)_2^{3-}$	−4.76	6.25 ± 0.17	6.13 ± 0.15	6.85 ± 0.17

Species	μ / 10^{-4} cm^2/(Vs)	log $K_{Li}^{0.335\ m}$	log $K_{Na}^{0.333\ m}$	log $K_{K}^{0.332\ m}$
$Me_2NpO_2(CO_3)_2^-$	−1.59	1.03 ± 0.19	1.01 ± 0.17	0.94 ± 0.15

Chemicals

Table S5 List of suppliers for the chemicals used in this work.

**Chemical**	**Supplier**	**Location**
Argon 4.6	Westfalen AG	Münster, Germany
Mili-Q-Water, 18,2 MΩ	Synergy^{TM}, Millipore GmbH	Schwalbach, Germany
ICP-MS-Standards Li, Mg, Co, Y, Ce, Tl	High-Purity Standards, Inc. (HPS)	Charleston, South Carolina, USA
Ethanol	VWR Chemicals BDH, VWR International	Radnor, Pennsylvania, USA
Nitric acid 65%	ChemSolute, Th. Geyer GmbH & Co. KG	Rennigen, Germany
Hydrochloric acid 37%	VWR Chemicals BDH, VWR International	Radnor, Pennsylvania, USA
Perchloric acid 70%	VWR Chemicals BDH, VWR International	Radnor, Pennsylvania, USA
Sodium chloride	VWR Chemicals BDH, VWR International	Radnor, Pennsylvania, USA
Sodium hydroxide	EMSURE, Merck KGaA	Darmstadt, Germany
Sodium carbonate	Merck KGaA	Darmstadt, Germany
Potassium chlorid	EMSURE, Merck KGaA	Darmstadt, Germany
Potassium carbonat	VWR Chemicals BDH, VWR International	Radnor, Pennsylvania, USA
Lithium chloride	Fluka Chemie AG	Buchs, Switzerland
Lithium carbonat	Acros Organics B.V.B.A.	Geel, Belgium
Cesium chloride	Merck KGaA	Darmstadt, Germany
Cesium carbonate	Thermo Fisher Scientific	Waltham, Massachusetts, USA
Rubidium chloride	Acros Organics	Geel, Flandern, Belgium
Rubidium carbonate	Thermo Fisher Scientific	Waltham, Massachusetts, USA
Hydroiodic acid	Merck KGaA	Darmstadt, Germany
Referenzpufferlösungen	SI Anlytics a Xylem brand	Mainz, Germany
2-Bromopropane	Merck KGaA	Darmstadt, Germany
MES	Merck KGaA	Darmstadt, Germany
HEPES	Carl Roth GmbH + Co. KG	Karlsruhe, Germany
CHES	Merck KGaA	Darmstadt, Germany

Ion Interaction Coefficients

Table S6. Ion interaction coefficients, ε(j,k) [kg·mol^{-1})] used in this work for cations/anions *j* with anions/cations *k*

j	*k*	ε(*j*,*k*)	Source
**Th^{4+}**	Cl^{-}	0.25	[11]
**Am^{3+}**	Cl^{-}	0.23	[7]
**$Th(OH)^{3+}$**	Cl^{-}	0.19	[9]
**$AmHCO_3^{2+}$**	Cl^{-}	0.16	[7]
**$Th(OH)_2^{2+}$**	Cl^{-}	0.13	[9]
**UO_2^{2+}**	Cl^{-}	0.21	[11]
**$AmCO_3^{+}$**	Cl^{-}	0.01	[3]
**H^{+}**	Cl^{-}	0.12	[11]
**NpO_2^{+}**	Cl^{-}	0.09	[13]
**$Th(OH)_3^{+}$**	Cl^{-}	0.06	[9]
**$Th(OH)(CO_3)^{+}$**	Cl^{-}	0.05	[12]
**$Am(CO_3)_2^{-}$**	Na^{+}	−0.14	[3]
**$NpO_2CO_3^{-}$**	Na^{+}	−0.18	[13]
**OH^{-}**	Na^{+}	0.04	[11]
**$Th(OH)(CO_3)_2^{-}$**	Na^{+}	−0.05	[12]
**$Th(OH)_3(CO_3)^{-}$**	Na^{+}	-0.05	[9]
**CO_3^{2-}**	Na^{+}	−0.08	[11]
**CO_3^{2-}**	K^{+}	−0.06	[11]
**$UO_2(CO_3)_2^{2-}$**	Na^{+}	−0.02	[11]
**$Th(OH)_2(CO_3)_2^{2-}$**	Na^{+}	−0.1	[9]
**$Th(OH)_4(CO_3)^{2-}$**	Na^{+}	−0.1	[9]
**$Am(CO_3)_3^{3-}$**	Na^{+}	−0.23	[3]
**$NpO_2(CO_3)_2^{3-}$**	Na^{+}	−0.33	[13]
**$UO_2(CO_3)_3^{4-}$**	Na^{+}	−0.01	[11]
**$NpO_2(CO_3)_2OH^{4-}$**	Na^{+}	−0.40	[11]
**$NpO_2(CO_3)_3^{5-}$**	Na^{+}	−0.53	[13]
**$Th(OH)(CO_3)_4^{5-}$**	Na^{+}	−0.3	[9]
**$Th(CO_3)_5^{6-}$**	Na^{+}	−0.3	[9]

Raw Data

Table S7. Sample composition for the determination of the complex formation constants in NaCl.

pH	$[CO_3^{2-}]$ / M	Buffer	$[\text{Buffer}_{-H}]$ / M	V_{HCl} / mL	[HCl] / M	m_{NaCl} / g	[NaCl] / M	I / M
1.34	7.70×10^{-15}	-	-	0.225	0.264	-	-	0.25
1.80	6.41×10^{-14}	-	-	0.190	0.224	0.0604	0.101	0.32
1.94	1.22×10^{-13}	-	-	0.185	0.218	0.0636	0.107	0.32
2.60	2.55×10^{-12}	-	-	0.180	0.212	0.0650	0.109	0.32
2.73	4.64×10^{-12}	-	-	0.179	0.211	0.0644	0.108	0.31
3.02	1.76×10^{-11}	-	-	0.178	0.210	0.0629	0.106	0.31
3.31	6.70×10^{-11}	MES	0.000	0.177	0.209	0.0670	0.113	0.32
3.73	4.62×10^{-10}	-	-	0.178	0.210	0.0618	0.104	0.31
4.10	2.52×10^{-9}	MES	0.001	0.179	0.211	0.0660	0.111	0.32
4.41	1.04×10^{-8}	MES	0.002	0.177	0.209	0.0630	0.106	0.31
4.84	7.28×10^{-8}	MES	0.005	0.175	0.206	0.0636	0.107	0.32
4.95	1.19×10^{-7}	MES	0.006	0.173	0.204	0.0657	0.111	0.32
4.97	1.30×10^{-7}	MES	0.006	0.173	0.204	0.0622	0.105	0.31
5.31	5.73×10^{-7}	MES	0.012	0.170	0.201	0.0554	0.093	0.31
5.61	1.00×10^{-6}	MES	0.019	0.165	0.195	0.0572	0.096	0.32
5.83	4.71×10^{-6}	MES	0.025	0.160	0.189	0.0564	0.095	0.32
5.84	4.89×10^{-6}	MES	0.026	0.159	0.188	0.0563	0.095	0.32
5.95	7.34×10^{-6}	MES	0.029	0.150	0.177	0.0494	0.083	0.31
6.75	9.95×10^{-5}	MES	0.045	0.100	0.119	0.0610	0.103	0.33
6.93	1.44×10^{-4}	HEPES	0.015	0.130	0.154	0.0689	0.116	0.34
7.13	2.39×10^{-4}	HEPES	0.021	0.095	0.113	0.0712	0.121	0.33
7.98	1.79×10^{-3}	HEPS	0.042	0.055	0.066	0.0663	0.113	0.32
8.29	3.61×10^{-3}	HEPES	0.046	0.075	0.089	0.0697	0.118	0.34
9.03	1.71×10^{-2}	CHES	0.025	0.055	0.066	0.0600	0.102	0.32
9.19	2.30×10^{-2}	CHES	0.030	0.055	0.066	0.0584	0.099	0.33
9.83	5.66×10^{-2}	CHES	0.043	-	-	0.0487	0.083	0.34
10.12	7.18×10^{-2}	CHES	0.046	0.060	0.072	0.0474	0.081	0.40
10.40	8.29×10^{-2}	CHES	0.048	-	-	0.0433	0.074	0.37
11.02	9.53×10^{-2}	-	-	-	-	0.0488	0.084	0.38
11.32	9.76×10^{-2}	-	-	-	-	0.0415	0.071	0.37
12.32*	9.98×10^{-2}	-	-	-	-	0.0237	0.041	0.35

* 0.005 M NaOH was added.

Table S8. Effective electrophoretic mobilities μ_{eff} [10^{-4} $cm^2/(Vs)$] of ^{241}Am, ^{232}Th, ^{237}Np, and ^{238}U in NaCl.

**pH**	**[CO_3^{2-}]**	**μ ^{241}Am**	**μ ^{232}Th**	**μ ^{237}Np**	**μ ^{238}U**	**Δμ**
1.34	7.70×10^{-15}	4.95	4.39*	2.35	3.30	0.04
1.80	6.41×10^{-14}	4.77	4.05*	2.28	3.21	0.04
1.94	1.22×10^{-13}	4.58	3.62*	2.08	3.04	0.04
2.60	2.55×10^{-12}	4.50	1.58*	2.14	2.98	0.04
2.73	4.64×10^{-12}	4.45	-	2.05	2.98	0.04
3.02	1.76×10^{-11}	4.45	-	2.09	2.81	0.04
3.31	6.70×10^{-11}	4.47	0.96*	2.05	2.80	0.03
3.73	4.62×10^{-10}	4.32	-	2.06	1.03	0.04
4.10	2.52×10^{-9}	4.06	-	2.10	−0.18	0.04
4.41	1.04×10^{-8}	3.84	-	2.06	−1.83	0.04
4.84	7.28×10^{-8}	3.38	-	2.01	−4.04	0.04
4.95	1.19×10^{-7}	3.27	−0.91	2.04	2.06 *	0.04
4.97	1.30×10^{-7}	2.99	-	2.03	−4.12	0.04
5.31	5.73×10^{-7}	2.27*	5.78 *	2.02	−3.90	0.04
5.61	1.00×10^{-6}	−2.74 *	−2.74	1.97	−2.74 *	0.04
5.83	4.71×10^{-6}	−1.55*	4.30 *	1.91	−4.39	0.04
5.84	4.89×10^{-6}	4.72 *	4.69 *	1.97	4.68 *	0.05
5.95	7.34×10^{-6}	-	-	1.75	-	-
6.75	9.95×10^{-5}	-	−4.51	−0.23	−4.07	0.05
6.93	1.44×10^{-4}	-	−4.40	−0.02*	−4.13	0.05
7.13	2.39×10^{-4}	-	-	−1.12	−4.03	0.06
7.98	1.79×10^{-3}	-	−5.03	−1.81	−3.93	0.06
8.29	3.61×10^{-3}	−2.55	−5.40	−1.67 *	−3.84	0.06
9.03	1.71×10^{-2}	-	−4.81	−2.79	−3.98	0.07
9.19	2.30×10^{-2}	−2.45	−4.97	−2.51 *	−3.95	0.07
9.83	5.66×10^{-2}	-	−4.25	−3.13	−4.00	0.07
10.12	7.18×10^{-2}	−2.76	−3.92	−3.04	−3.94	0.07
10.40	8.29×10^{-2}	−2.97	−4.43	−3.18	−4.03	0.07
11.02	9.53×10^{-2}	-	−1.63 *	−3.52	−4.33	0.07
11.32	9.76×10^{-2}	−3.38 *	−1.91 *	−3.48	−4.30	0.07
12.32	9.98×10^{-2}	-	3.29 *	−3.59	−4.21	0.07

* Outliers, not considered in the fit.

Table S9. Sample composition for the determination of the complex formation constants in LiCl.

pH	$[CO_3^{2-}]$ / M	Buffer	$[Buffer_{-H}]$ / M	V_{HCl} / mL	[HCl] / M	m_{LiCl} / g	[LiCl] / M	*I* / M
1.67	3.52×10^{-14}	-	-	0.190	0.224	0.0425	0.098	0.32
2.37	8.84×10^{-13}	-	-	0.186	0.219	0.0438	0.101	0.31
2.96	1.34×10^{-11}	-	-	0.184	0.217	0.0463	0.107	0.32
3.23	4.63×10^{-11}	MES	0.000	0.185	0.218	0.0453	0.105	0.31
4.16	3.32×10^{-9}	MES	0.001	0.182	0.214	0.0452	0.105	0.31
4.45	1.25×10^{-8}	MES	0.002	0.181	0.213	0.0471	0.109	0.32
4.64	2.96×10^{-8}	MES	0.003	0.179	0.211	0.0453	0.105	0.31
5.03	1.70×10^{-7}	MES	0.007	0.174	0.205	0.0388	0.090	0.30
5.31	5.73×10^{-7}	MES	0.012	0.170	0.201	0.0449	0.104	0.32
5.60	1.92×10^{-6}	MES	0.019	0.165	0.195	0.0406	0.094	0.31
5.93	6.82×10^{-6}	MES	0.028	0.150	0.177	0.0426	0.099	0.32
6.17	1.58×10^{-5}	MES	0.035	0.135	0.160	0.0494	0.115	0.34
6.74	8.71×10^{-5}	MES	0.045	0.100	0.119	0.0426	0.099	0.32
7.25	3.21×10^{-4}	HEPES	0.024	0.090	0.107	0.0540	0.126	0.34
8.29	3.61×10^{-3}	HEPES	0.046	0.050	0.060	0.0551	0.129	0.34
9.08	1.88×10^{-2}	CHES	0.026	0.050	0.060	0.0600	0.141	0.36
9.87	5.89×10^{-2}	CHES	0.044	-	-	0.0417	0.098	0.36
11.02	9.53×10^{-2}	-	-	-	-	0.0349	0.082	0.38

Table S10. Effective electrophoretic mobilities μ_{eff} $[10^{-4}\ cm^2/(Vs)]$ of ^{241}Am, ^{232}Th, ^{237}Np, and ^{238}U in LiCl.

pH	$[CO_3^{2-}]$	μ ^{241}Am	μ ^{232}Th	μ ^{237}Np	μ ^{238}U	$\Delta\mu$
1.67	3.52×10^{-14}	4.57	3.7	2.16	3.11	0.04
2.37	8.84×10^{-13}	4.38	3.21	2.00	2.86	0.04
2.96	1.34×10^{-11}	4.26	1.18	1.98	2.40*	0.03
3.23	4.63×10^{-11}	4.22	2.09	1.98	2.55	0.03
4.16	3.32×10^{-9}	4.06	−1.44	2.04	−1.03	0.03
4.45	1.25×10^{-8}	3.14	−1.85	1.99	−1.40	0.04
4.64	2.96×10^{-8}	2.86	−3.69	1.97	−2.90	0.04
5.03	1.70×10^{-7}	−0.14	-	1.93	-	0.04
5.31	5.73×10^{-7}	-	-	1.86	-	0.04
5.60	1.92×10^{-6}	-	3.93	1.84	-	0.05
5.93	6.82×10^{-6}	-	4.06	1.59	-	0.05
6.17	1.58×10^{-5}	-	-	1.34	-	0.05
6.74	8.71×10^{-5}	-	-	−0.39	−4.11	0.05
7.25	3.21×10^{-4}	-	-	−1.31	−3.95	0.06
8.29	3.61×10^{-3}	-	-	−2.12	−3.88	0.07
9.08	1.88×10^{-2}	-	−4.03	−2.81	−4.56*	0.07
9.87	5.89×10^{-2}	-	−3.91	−3.04	−4.00	0.07
11.02	9.53×10^{-2}	-	−1.88	−3.44	−4.38*	0.07

* Outliers, not considered in the fit.

Table S11. Sample composition for the determination of the complex formation constants in KCl.

pH	$[CO_3^{2-}]$ / M	Buffer	$[Buffer_{-H}]$ / M	V_{HCl} / mL	[HCl] / M	m_{KCl} / g	[KCl] / M	I / M
1.65	2.91×10^{-14}	-	-	0.185	0.218	0.0773	0.102	0.32
2.58	2.10×10^{-12}	-	-	0.170	0.201	0.0886	0.117	0.32
2.94	1.10×10^{-11}	-	-	0.169	0.199	0.0896	0.118	0.32
3.85	7.25×10^{-10}	MES	0.001	0.168	0.198	0.0911	0.120	0.32
4.15	2.87×10^{-9}	MES	0.001	0.167	0.197	0.0874	0.115	0.32
4.56	1.86×10^{-8}	MES	0.003	0.166	0.196	0.0850	0.112	0.31
4.59	2.13×10^{-8}	MES	0.003	0.164	0.194	0.0901	0.119	0.32
4.92	9.40×10^{-8}	MES	0.006	0.168	0.198	0.0896	0.118	0.32
5.25	3.99×10^{-7}	MES	0.011	0.155	0.183	0.0875	0.116	0.32
5.31	5.16×10^{-7}	MES	0.012	0.161	0.190	0.0805	0.106	0.31
5.62	1.86×10^{-6}	MES	0.019	0.150	0.177	0.0808	0.107	0.32
5.85	4.52×10^{-6}	MES	0.026	0.140	0.166	0.0840	0.111	0.33
6.30	2.12×10^{-5}	MES	0.038	0.120	0.142	0.0812	0.108	0.33
6.92	1.23×10^{-4}	MES	0.046	0.080	0.095	0.0872	0.116	0.33
7.38	3.83×10^{-4}	HEPES	0.028	0.075	0.089	0.0939	0.125	0.33
8.05	1.83×10^{-3}	HEPES	0.043	0.045	0.054	0.0991	0.132	0.33
9.11	1.78×10^{-2}	CHES	0.027	0.045	0.054	0.0866	0.116	0.33
9.84	5.37×10^{-2}	CHES	0.043	-	-	0.0594	0.080	0.33
10.89	9.29×10^{-2}	-	-	-	-	0.0578	0.078	0.37

Table S12. Effective electrophoretic mobilities μ_{eff} [10^{-4} $cm^2/(Vs)$] of ^{241}Am, ^{232}Th, ^{237}Np, and ^{238}U in KCl.

pH	$[CO_3^{2-}]$	μ ^{241}Am	μ ^{232}Th	μ ^{237}Np	μ ^{238}U	$\Delta\mu$
1.65	2.91×10^{-14}	4.99	4.16	2.29	3.44	0.04
2.58	2.10×10^{-12}	4.95	0.25	2.28	3.19	0.03
2.94	1.10×10^{-11}	4.87	-	2.26	2.95	0.03
3.85	7.25×10^{-10}	4.68	-	2.31	0.23	0.03
4.15	2.87×10^{-9}	4.69	-	2.27	−1.58*	0.03
4.56	1.86×10^{-8}	4.39	−2.94	2.26	−2.08	0.03
4.59	2.13×10^{-8}	4.19	-	-	−1.10*	0.03
4.92	9.40×10^{-8}	-	-	2.62	−3.57	0.03
5.25	3.99×10^{-7}	1.96	-	1.92	-	0.04
5.31	5.16×10^{-7}	-	-	2.20	-	0.04
5.62	1.86×10^{-6}	-	-	2.06	-	0.04
5.85	4.52×10^{-6}	-	-	1.83	-	0.04
6.30	2.12×10^{-5}	-	-	1.29	-	0.04
6.92	1.23×10^{-4}	-	-	−0.84	−4.56	0.05
7.38	3.83×10^{-4}	-	-	−1.45	−4.40	0.06
8.05	1.83×10^{-3}	-	-	−2.52	−4.38	0.06
9.11	1.78×10^{-2}	-	-	−3.24	−4.33	0.07
9.84	5.37×10^{-2}	-	-	−3.68	−4.31	0.07

* Outliers, not considered in the fit.

Table S13 Sample composition for the investigation of the alkali association and effective electrophoretic mobilities of ^{127}I and ^{238}U. Due to high ^{127}I background in KCl samples, no electrophoretic mobility of I^- was measured.

I / M	pH	[LiCl] / M	[Li_2CO_3] / M	μ ^{127}I / 10^{-4} $cm^2/(Vs)$	μ ^{238}U / 10^{-4} $cm^2/(Vs)$
0.05	10.85	0.035	0.005	−6.93 ± 0.2	−5.29 ± 0.2
0.10	10.68	0.087	0.005	−6.84 ± 0.1	−4.71 ± 0.1
0.15	10.65	0.136	0.005	−6.82 ± 0.1	−4.48 ± 0.1
0.20	10.44	0.184	0.005	−6.87 ± 0.1	−4.33 ± 0.1
0.25	10.39	0.237	0.005	−6.92 ± 0.1	−4.07 ± 0.1
0.30	10.31	0.285	0.005	−7.05 ± 0.1	−4.14 ± 0.1
***I* / M**	**pH**	**[NaCl] / M**	**[Na_2CO_3] / M**		
0.05	10.64	0.035	0.005	−6.91 ± 0.2	−5.31 ± 0.2
0.10	10.51	0.085	0.005	−6.89 ± 0.1	−4.86 ± 0.1
0.15	10.42	0.135	0.005	−6.93 ± 0.1	−4.57± 0.1
0.20	10.34	0.185	0.005	−7.01 ± 0.1	−4.35± 0.1
0.25	10.29	0.234	0.005	−7.09 ± 0.1	−4.34± 0.1
0.30	10.26	0.285	0.005	−6.97 ± 0.1	−4.38± 0.1
***I* / M**	**pH**	**[KCl] / M**	**[K_2CO_3] / M**		
0.05	10.68	0.037	0.005	-	−5.27 ± 0.2
0.10	10.60	0.084	0.005	-	−4.82 ± 0.1
0.15	10.54	0.136	0.005	-	−4.72 ± 0.1
0.20	10.48	0.185	0.005	-	−4.59 ± 0.1
0.25	10.44	0.236	0.005	-	−4.56 ± 0.1
0.30	10.41	0.285	0.005	-	−4.60 ± 0.2
***I* / M**	**pH**	**[RbCl] / M**	**[Rb_2CO_3] / M**		
0.05	10.83	0.036	0.005	−6.89 ± 0.2	−5.20 ± 0.2
0.10	10.70	0.085	0.005	−6.89 ± 0.2	−4.96 ± 0.2
0.15	10.70	0.136	0.005	−6.93 ± 0.2	−5.09 ± 0.2
0.20	10.67	0.185	0.005	-	-
0.25	10.67	0.235	0.005	−7.09 ± 0.2	−4.87 ± 0.2
0.30	10.61	0.285	0.005	-	−4.92 ± 0.2
***I* / M**	**pH**	**[CsCl] / M**	**[Cs_2CO_3] / M**		
0.05	10.87	0.034	0.005	−6.91 ± 0.2	−5.21 ± 0.2
0.10	10.77	0.085	0.005	−6.84 ± 0.2	−4.59* ± 0.1
0.15	10.72	0.135	0.005	−6.87 ± 0.2	−4.53*± 0.1
0.20	10.68	0.185	0.005	−7.02 ± 0.2	−4.75 ± 0.2
0.25	10.64	0.235	0.005	-	−4.85 ± 0.2
0.30	10.62	0.285	0.005	-	−4.77 ± 0.2

* Outliers, not considered in the fit.

Electropherograms

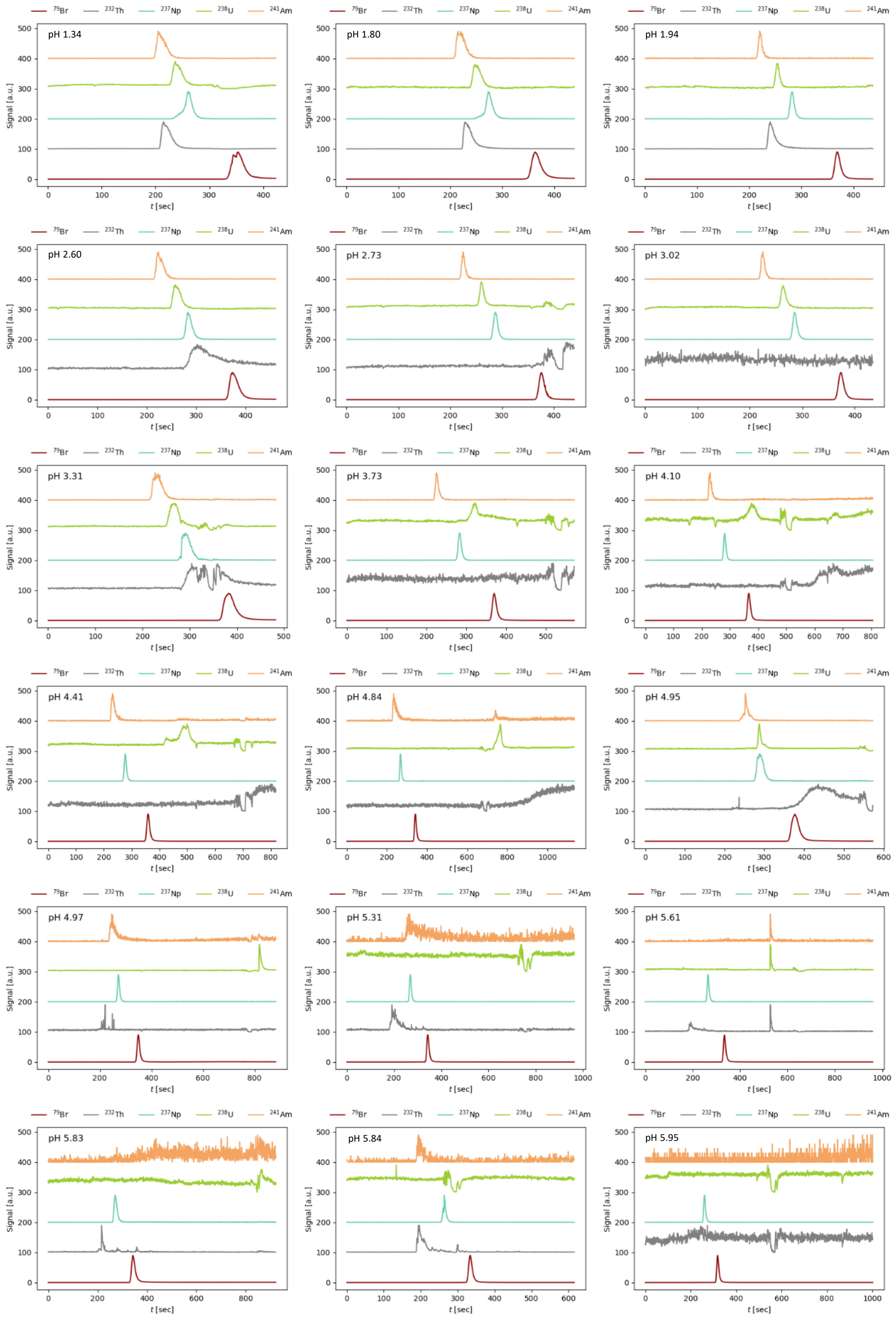

Figure S11. Electropherograms of ^{232}Th, ^{237}Np, ^{238}U, ^{241}Am, and ^{79}Br (EOF) in 0.1 M Na_2CO_3 solution, I = 0.333 m (NaCl), varied pH, 25 °C, normalized signal, l = 50 cm, measured at 10 kV.

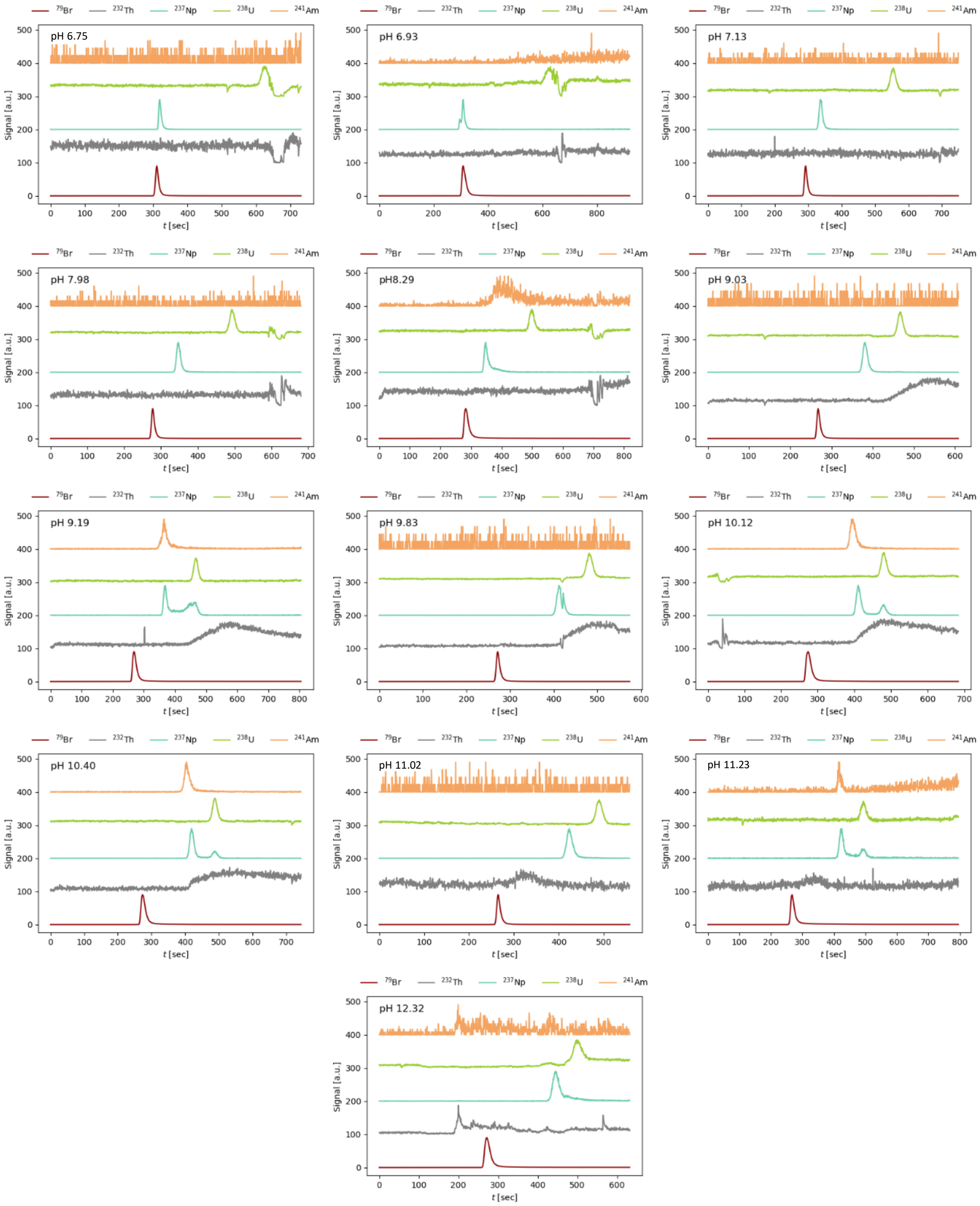

Figure S12 Continuation of Figure S11: Electropherograms of ^{232}Th, ^{237}Np, ^{238}U, ^{241}Am, and ^{79}Br (EOF) in 0.1 M Na_2CO_3 solution, I = 0.333 m (NaCl), varied pH, 25 °C, normalized signal, l = 50 cm, measured at 10 kV.

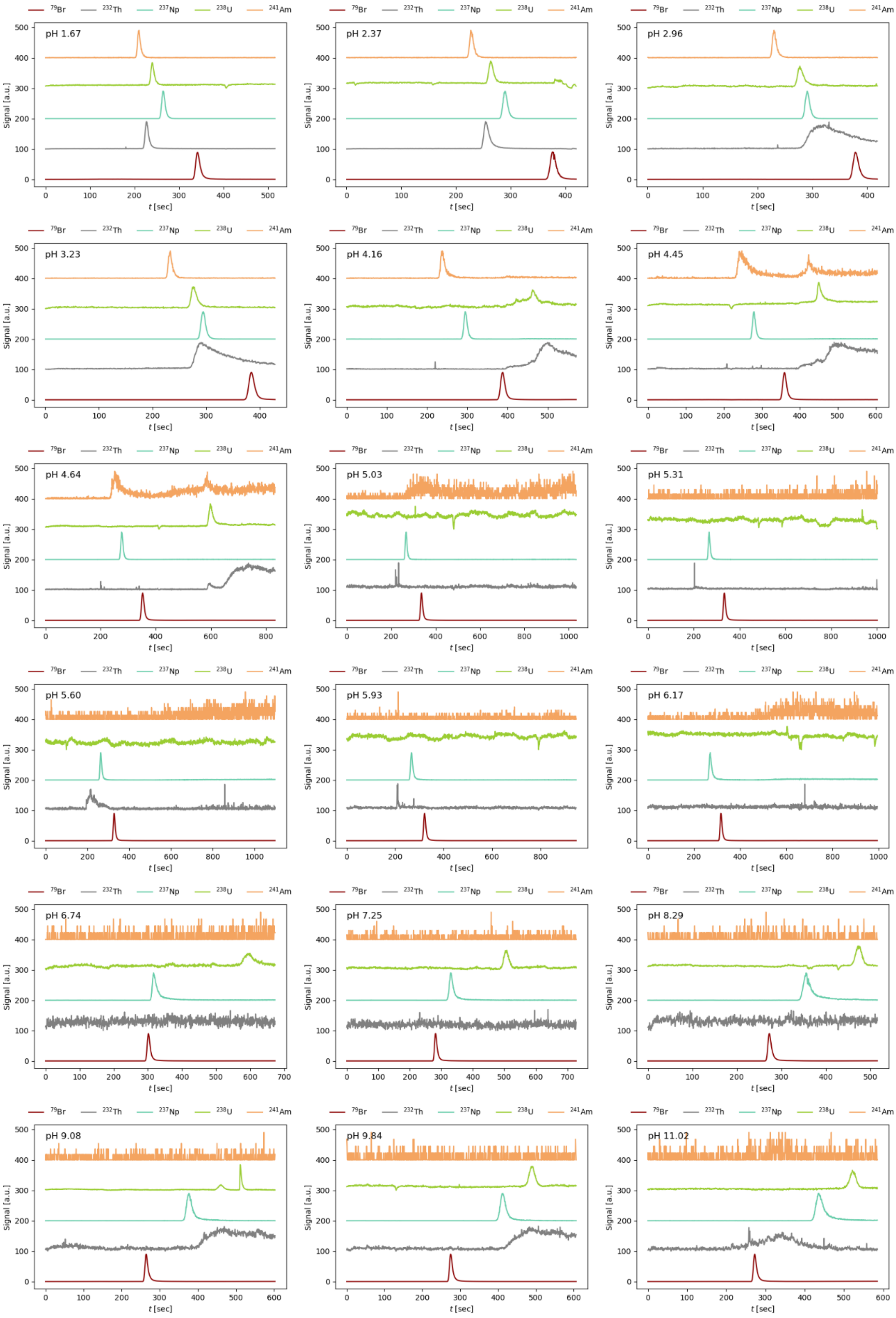

Figure S13. Electropherograms of ^{232}Th, ^{237}Np, ^{238}U, ^{241}Am, and ^{79}Br (EOF) in 0.1 M Li_2CO_3 solution, I = 0.335 m (LiCl), varied pH, 25 °C, normalized signal, l = 50 cm, measured at 10 kV.

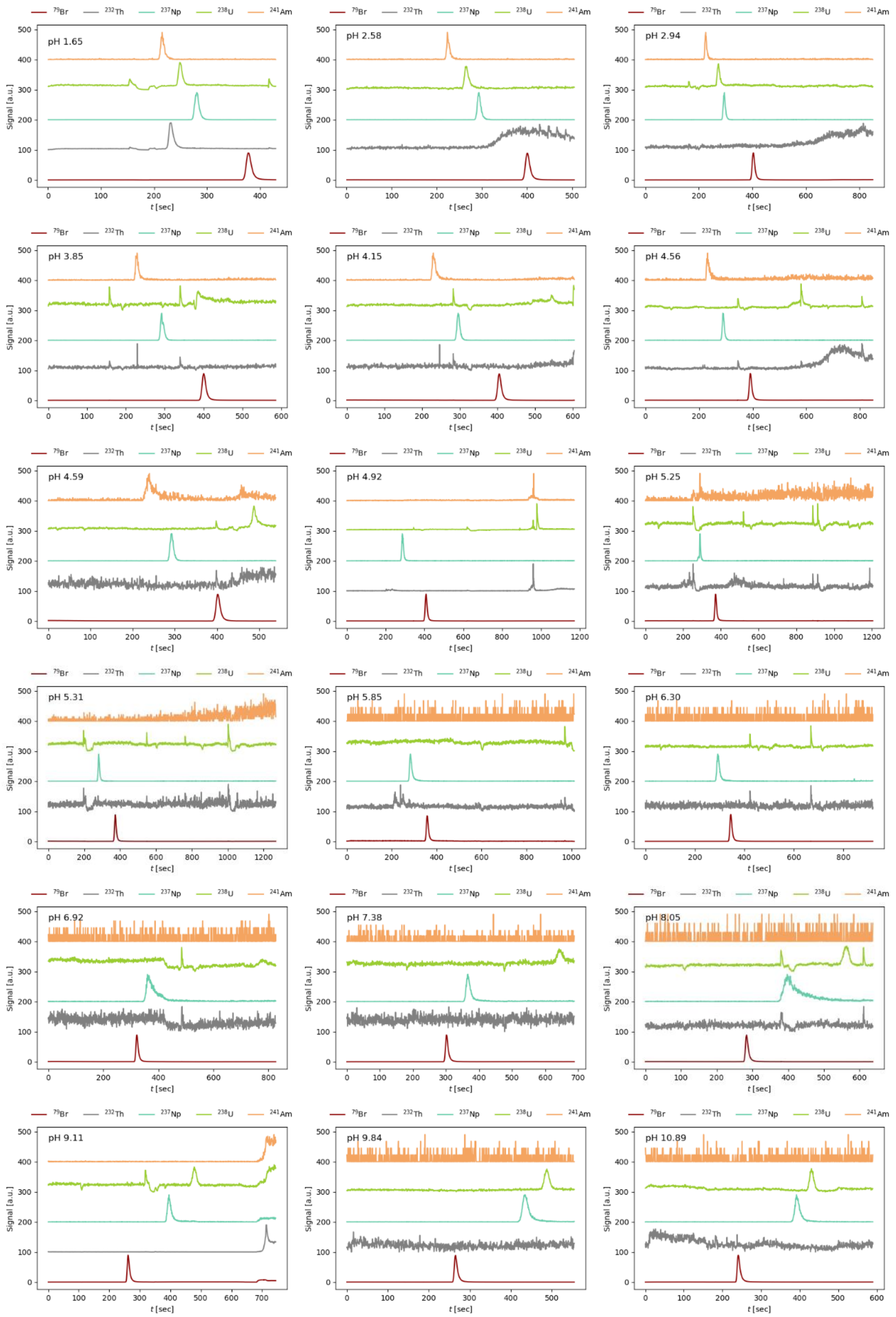

Figure S14. Electropherograms of ^{232}Th, ^{237}Np, ^{238}U, ^{241}Am, and ^{79}Br (EOF) in 0.1 M K_2CO_3 solution, I = 0.332 m (KCl), varied pH, 25 °C, normalized signal, l = 50 cm, measured at 10 kV.

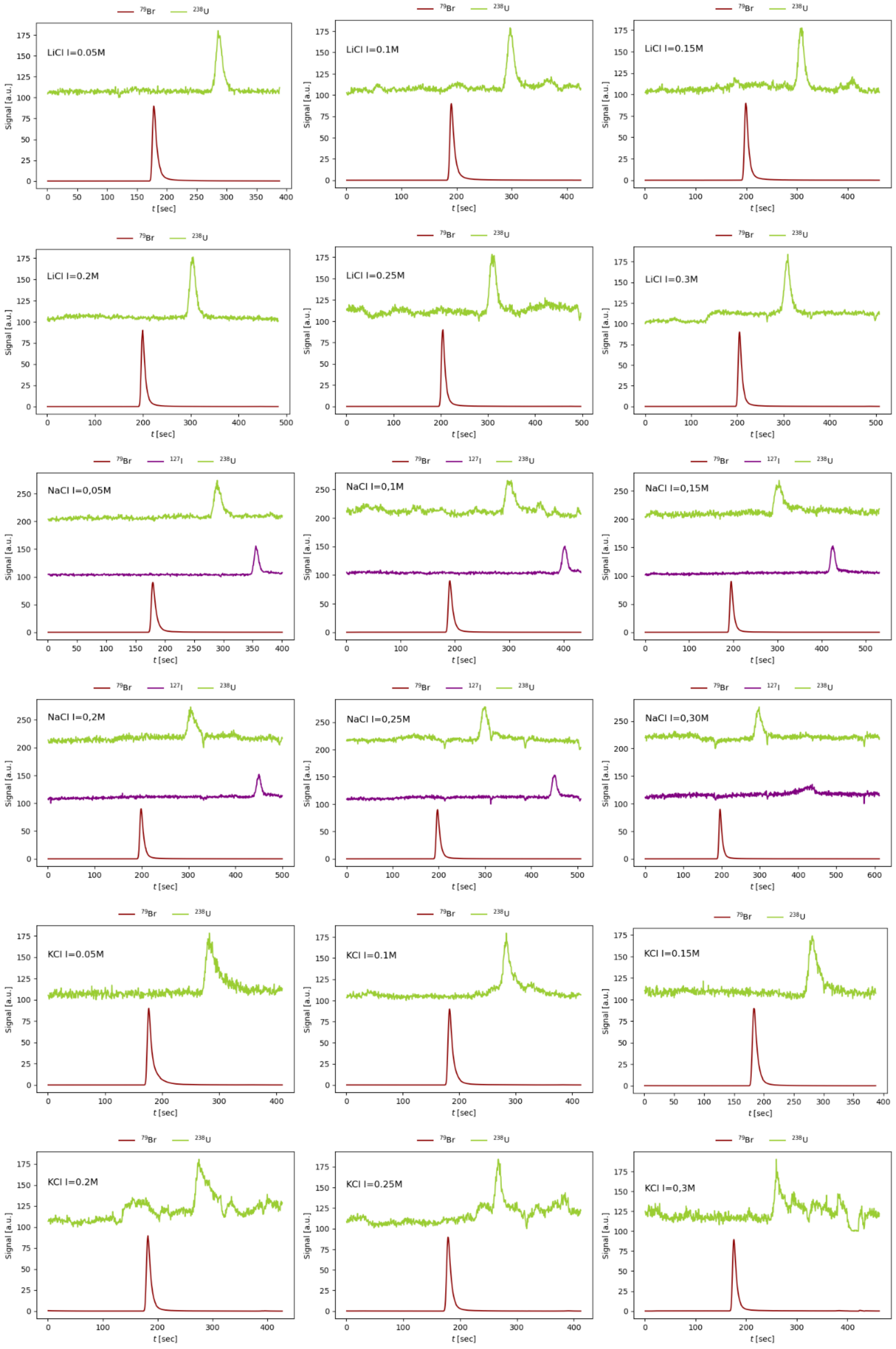

Figure S15. Electropherograms of ^{127}I, ^{238}U, and ^{79}Br (EOF) in 0.01 M $(Li/Na/K)_2CO_3$ solution, varied ionic strength (LiCl, NaCl, KCl), 25 °C, normalized signal, l = 50 cm, measured at 10 kV.

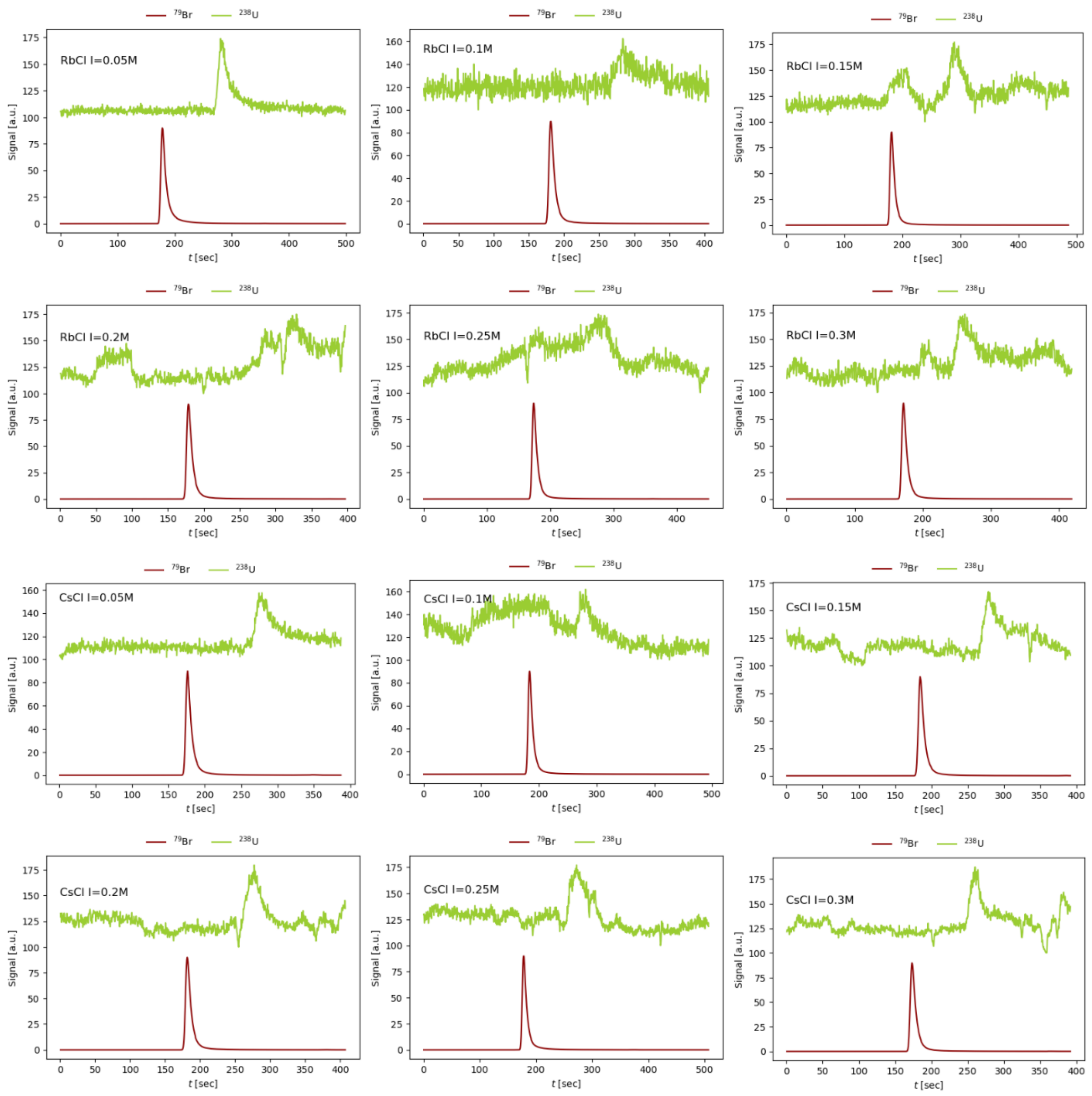

Figure S16. Electropherograms of ^{238}U and ^{79}Br (EOF) in 0.01 M $(Rb/Cs)_2CO_3$ solution, varied ionic strength (RbCl, CsCl), 25 °C, normalized signal, l = 50 cm, measured at 10 kV.

Correlation matrices

Unreported correlations are < 0.100.

Figure 1-main

Am(III)
C(β1, β3) = 0.625
C(β1, βH1) = −0.518
C(β3, βH1) = −0.324

Np(V)
C(β3, β2OH) = −0.594
C(β2, β2OH) = 0.465
C(β2, β3) = 0.247

U(VI)
C(β2, β3) = −0.685
C(β1, β3) = 0.625
C(β1, β2) = −0.159

Figure 4-main

	Li^+	Na^+	K^+	Rb^+	Cs^+
C(K1, K2)	−0.999	−0.996	−0.997	−0.922	−0.939

References

(1) R. Guillaumont, T. Fanghänel, J. Fuger, I. Grenthe, V. Neck, D. A. Palmer, and M. H. Rand. *Update on the Chemical Thermodynamics of Uranium, Neptunium, Plutonium, Americium and Technetium*; Chemical Thermodynamics, Vol. 5; OECD Publishing, 2003.

(2) Mandal, P.; Kretzschmar, J.; Drobot, B. Not Just a Background: pH Buffers Do Interact with Lanthanide Ions-A Europium(III) Case Study. *J. Biol. Inorg. Chem.* **2022**, *27* (2), 249–260. DOI: 10.1007/s00775-022-01930-x. Published Online: Feb. 12, 2022.

(3) I. Grenthe, X. Gaona, A. V. Plyasunov, L. Rao, W. H. Runde, B. Grambow, R. J. M. Konings, A. L. Smith, and E. E. Moore. *Second Update on the Chemical Thermodynamics of U, Np, Pu, Am and Tc*; Chemical Thermodynamics, Vol. 14; OECD Publishing, 2020.

(4) Fanghänel, T.; Könnecke, T.; Weger, H.; Paviet-Hartmann, P.; Neck, V.; Kim, J. I. Thermodynamics of Cm(III) in Concentrated Salt Solutions: Carbonate Complexation in NaCl Solution at 25°C. *J. Solution Chem.* **1999**, *28* (4), 447–462. DOI: 10.1023/A:1022664013648.

(5) D.L. Parkhurst, C. A. *Description of Input and Examples for PHREEQC Version 3-A Computer Program for Speciation, Batch-Reaction, One-Dimensional Transport, and Inverse Geochemical Calculations*; U.S. Geological Survey Techniques and Methods, Vol. 6, 2013.

(6) Giffaut, E.; Grivé, M.; Blanc, P.; Vieillard, P.; Colàs, E.; Gailhanou, H.; Gaboreau, S.; Marty, N.; Madé, B.; Duro, L. Andra Thermodynamic Database for Performance Assessment: ThermoChimie. *Appl. Geochem.* **2014**, *49*, 225–236. DOI: 10.1016/j.apgeochem.2014.05.007.

(7) David G. Kinniburgh; David Cooper. PhreePlot: Creating Graphical Output with PHREEQC. In.

(8) Altmaier, M.; Neck, V.; Denecke, M. A.; Yin, R.; Fanghänel, T. Solubility of $ThO_2 \cdot xH_2O$(am) and the Formation of Ternary Th(IV) Hydroxide-Carbonate Complexes in $NaHCO_3$-Na_2CO_3 Solutions Containing 0−4 M NaCl. *Radiochim. Acta* **2006**, *94* (9-11), 495–500. DOI: 10.1524/ract.2006.94.9-11.495.

(9) M. Rand, J. Fuger, I. Grenthe, V. Neck and D. Rai. *Chemical Thermodynamics of Thorium*; Chemical Thermodynamics, Vol. 11; OECD Publishing, 2009.

(10) Shannon, R. D. Revised Effective Ionic Radii and Systematic Studies of Interatomic Distances in Halides and Chalcogenides. *Acta Cryst A* **1976**, *32* (5), 751–767. DOI: 10.1107/S0567739476001551.

(11) I. Grenthe, F. Mompean, K. Spahiu, and H. Wanner. *Guidelines for the Extrapolation to Zero Ionic Strength*, Vol. 2; OECD Publishing, 2013.

(12) Altmaier, M.; Neck, V.; Müller, R.; Fanghänel, T. Solubility of $ThO_2 \cdot xH_2O$(am) in Carbonate Solution and the Formation of Ternary Th(IV) Hydroxide-Carbonate Complexes. *Radiochim. Acta* **2005**, *93* (2), 83–92. DOI: 10.1524/ract.93.2.83.59420.

(13) R. J. Lemire, J. Fuger, H. Nitsche, P. Potter, M. H. Rand, J. Rydberg, K. Spahiu, J. C. Sullivan, W. J. Ullman, P. Vitorge, and H. Wanner. *Chemical Thermodynamics of Neptunium and Plutonium*; Chemical Thermodynamics, Vol. 4; OECD Publishing, 2001.
